# Comparative analyses of vertebrate CPEB proteins define two subfamilies with coordinated yet distinct functions in post-transcriptional gene regulation

**DOI:** 10.1186/s13059-022-02759-y

**Published:** 2022-09-12

**Authors:** Berta Duran-Arqué, Manuel Cañete, Chiara Lara Castellazzi, Anna Bartomeu, Anna Ferrer-Caelles, Oscar Reina, Adrià Caballé, Marina Gay, Gianluca Arauz-Garofalo, Eulalia Belloc, Raúl Mendez

**Affiliations:** 1 Institute for Research in Biomedicine (IRB Barcelona), The Barcelona Institute of Science and Technology, 08028 Barcelona, Spain; 2 Institució Catalana de Recerca I Estudis Avançats (ICREA), 08010 Barcelona, Spain

**Keywords:** mRNA translation, Deadenylation, 3′ UTR, BioID, Phase separation, Phosphorylation, CCR4-NOT complex, CPEB

## Abstract

**Background:**

Vertebrate CPEB proteins bind mRNAs at cytoplasmic polyadenylation elements (CPEs) in their 3′ UTRs, leading to cytoplasmic changes in their poly(A) tail lengths; this can promote translational repression or activation of the mRNA. However, neither the regulation nor the mechanisms of action of the CPEB family per se have been systematically addressed to date.

**Results:**

Based on a comparative analysis of the four vertebrate CPEBs, we determine their differential regulation by phosphorylation, the composition and properties of their supramolecular assemblies, and their target mRNAs. We show that all four CPEBs are able to recruit the CCR4-NOT deadenylation complex to repress the translation. However, their regulation, mechanism of action, and target mRNAs define two subfamilies. Thus, CPEB1 forms ribonucleoprotein complexes that are remodeled upon a single phosphorylation event and are associated with mRNAs containing canonical CPEs. CPEB2–4 are regulated by multiple proline-directed phosphorylations that control their liquid–liquid phase separation. CPEB2–4 mRNA targets include CPEB1-bound transcripts, with canonical CPEs, but also a specific subset of mRNAs with non-canonical CPEs.

**Conclusions:**

Altogether, these results show how, globally, the CPEB family of proteins is able to integrate cellular cues to generate a fine-tuned adaptive response in gene expression regulation through the coordinated actions of all four members.

**Supplementary Information:**

The online version contains supplementary material available at 10.1186/s13059-022-02759-y.

## Background

An estimated 20–30% of all vertebrate genes are regulated by the cytoplasmic polyadenylation elements (CPEs) that are present in the 3′ UTR of their transcripts [1, 2]. These *cis*-acting elements recruit members of the CPE-binding protein (CPEB) family of RNA-binding proteins. In turn, CPEBs regulate mRNA translation, either by assembling repressor complexes that maintain target transcripts translationally silenced or by promoting cytoplasmic polyadenylation and subsequent translational activation [3, 4]. This family of proteins appears to have evolved by gene duplication and divergence (Additional file 1: Fig. S1A). Thus, *Aplysia* has only one CPEB (ApCPEB), *Drosophila* has two (Orb and Orb2), and all vertebrates have four (CPEB1–4). The “primitive” CPEB appears to be CPEB1/ApCPEB/Orb, whereas CPEB2–4 are more similar between themselves and to Orb2. All four vertebrate CPEBs are differentially expressed in somatic tissues but with overlapping patterns. Thus, multiple CPEBs co-exist in individual cells (Additional file 1: Fig. S1B), potentially co-regulating overlapping populations of transcripts. CPEBs share similar C-terminal RNA-binding domains, comprising two RNA recognition motifs (RRMs) in tandem, followed by a ZZ domain (a zinc-binding domain with a cross-braced zinc binding topology). However, the structures of these regions, both in the free state and bound to a CPE, revealed that recognition of the CPE by CPEBs is different between CPEB1 and CPEB2-4 [5]. The CPEB N-terminal domain (NTD) is highly variable both in length and composition across various CPEB orthologs and paralogs [6] and contains intrinsically disordered regions (IDR), which are more extended in CPEB2–4 [7] (Additional file 1: Fig. S1C). For CPEB1 and CPEB4, the switch from repression to activation is differentially regulated through post-translational modifications in the NTD. Specifically, CPEB1 is regulated by two sequential phosphorylation events. First, Aurora kinase A (AurKA) phosphorylates CPEB1 at S174, promoting the switch from a repressor to an activator. Subsequently, Cdk1 and Polo-like kinase 1 (Plk1) target CPEB1 NTD to ubiquitin-mediated degradation [8, 9, 10]. CPEB4 is activated by ERK2- and Cdk1-mediated phosphorylation at 12 residues in CPEB4’s NTD [7]. These regulatory posttranslational modifications of CPEBs have been linked to either remodeling of the CPEB-ribonucleoprotein complex (CPEB-mRNP) or with cell cycle phase transitions. For CPEB1, AurKA promotes the dissociation of the repressor complex, although three different and mutually incompatible models have been proposed [4]. CPEB4 regulation during the cell cycle has been linked to phase transitions [7]. In neurons, CPEB3, Orb2, and ApCPEB are regulated by mono-ubiquitination and SUMOylation [11, 12], but the targeted residues have not been identified. How CPEB2 and CPEB3 are regulated during the cell cycle or by phosphorylation has not been addressed. Even though it is clear that CPEB1 and CPEB4 bind overlapping populations of target mRNAs [5, 13, 14, 15, 16], they perform differential functions, as indicated by the paralog-specific phenotypes when individually depleted [15, 17, 18, 19, 20, 21, 22]. Moreover, it appears that their functions/activities may be somehow coordinated, at least temporarily during the cell cycle [7, 13, 14, 15].

Because most studies so far have addressed individual CPEB proteins, it remains unclear whether the four vertebrate CPEB family members assemble similar or distinct repressor complexes, are subject to differential regulation, and display distinct target specificities. Conducting such a comparative analysis is essential to understand the regulation of CPE-containing mRNAs in cells/tissues that co-express multiple CPEBs and gain insight into the evolutionary expansion of CPEB family members and their possible functional specialization. Here, we perform a systematic comparative analysis of the four vertebrate CPEBs and reveal key differences in their regulation by phosphorylation, the composition and properties of their supramolecular assemblies, and their mRNA targets.

## Results

### All CPEBs are expressed and phosphorylated in the meiotic cell cycle

**Fig. 1:**
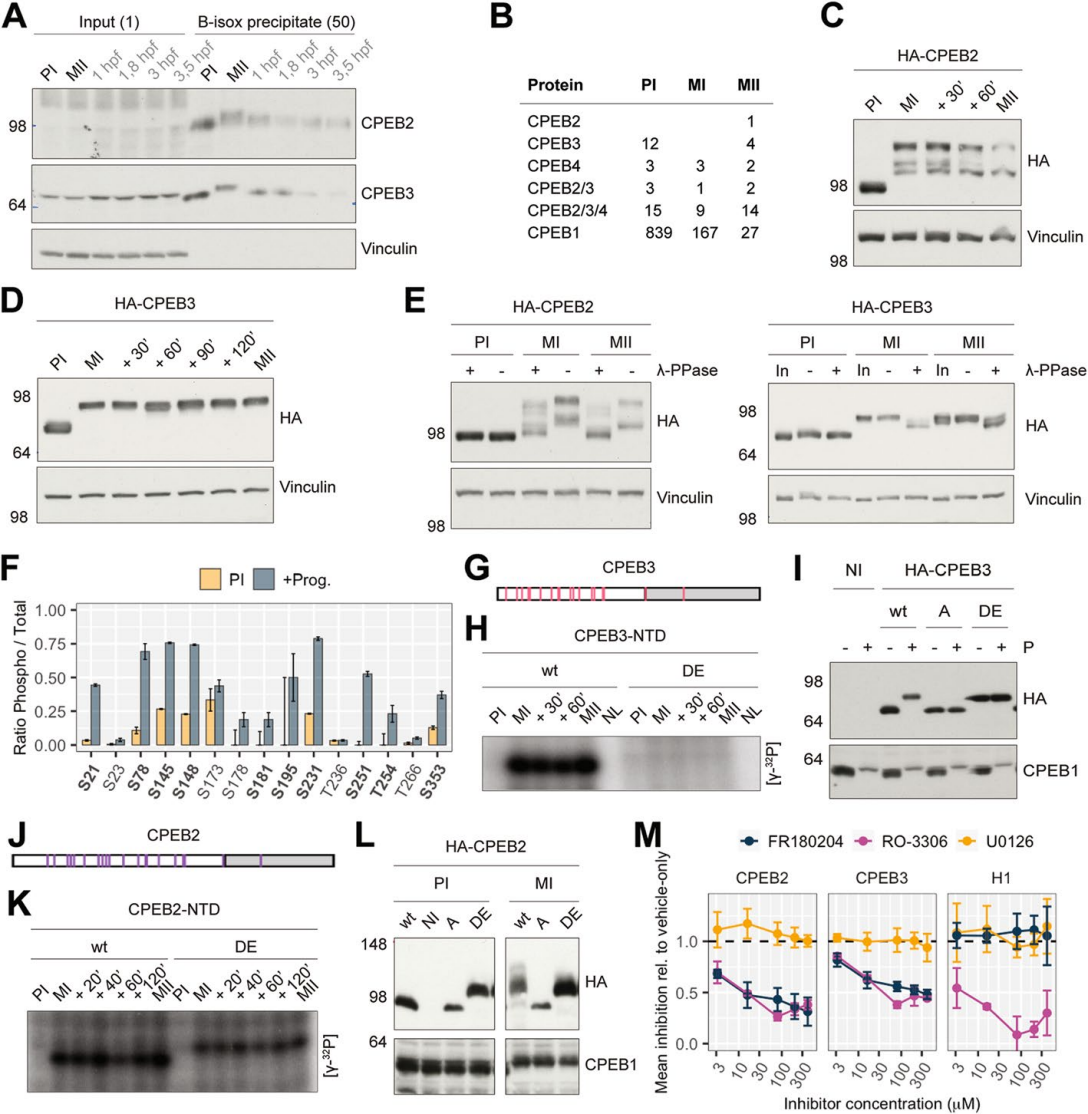
CPEBs are co-expressed and co-regulated by phosphorylation in the meiotic cell cycle. **A** Endogenous CPEB2 and CPEB3 immunoblots from b-isox-precipitated extracts from the indicated meiotic maturation and early development time points. CPEB2, *n* = 2; CPEB3, *n* = 4. Vinculin immunoblot was used as a loading control. The number of oocytes loaded is indicated in parentheses. **B** Table showing the number of peptide spectrum matches (PSMs) of the CPEBs detected by MS/MS after b-isox-precipitation of meiotic maturation lysates, *n* = 1. **C**, **D**. Western blots of HA-CPEB2 (*n* = 2) and HA-CPEB3 (*n* = 2) overexpressing oocytes during meiotic maturation. Vinculin immunoblot was used as a loading control. **E** Lambda phosphatase assays (λ-PPase) of HA-CPEB2 (*n* = 2) and HA-CPEB3 (*n* = 2) overexpressing oocytes at the indicated time points. Western blots of anti-HA and anti-vinculin are shown. **F** CPEB3 phospho-to-total occurrence ratios determined by MS/MS for the indicated residue positions on prophase I (PI) versus progesterone-treated oocytes (+ Prog.). The ratios were calculated from the pool of 4 biological replicates. Only positions with a relative gain of phosphorylation upon progesterone treatment are displayed. Proline-directed sites are highlighted in bold. Error bars represent the ratio error. **G** Relative positions of the 18 proline-directed sites in CPEB3. The NTD is white-shaded, whereas the CTD is gray-shaded. **H** Wild-type (*wt*) and phosphomimetic (DE) CPEB3-NTD [γ-32P]-ATP incorporation upon incubation with oocyte lysates at the indicated maturation time points (*n* = 2). **I** Western blot against HA-tag and endogenous CPEB1 of stage VI ( −) and progesterone stimulated oocytes ( +) overexpressing wild-type HA-CPEB3 (*wt*), phosphonull HA-CPEB3 (A), and phosphomimetic HA-CPEB3 (DE). Not injected (NI) (*n* = 6). **J** Relative positions of the 20 proline-directed sites in CPEB2. The NTD is white-shaded, whereas the CTD is gray-shaded. **K** [γ-32P]-ATP incorporation by wild-type (*wt*) or phosphomutant (DE) CPEB2-NTD upon incubation with oocyte lysates at the indicated maturation time points (*n* = 3). **L** Western blot against HA-tag and CPEB1 of stage VI ( −) and progesterone stimulated oocytes ( +) overexpressing wild-type HA-CPEB2 (*wt*), not injected (NI), phosphonull HA-CPEB2 (A), and phosphomimetic HA-CPEB2 (DE) (*n* = 3). **M** The mean inhibition of [γ-32P]-ATP incorporation to CPEB2-NTD, CPEB3-NTD, or Histone H1 (H1) by increasing inhibitor concentrations. Data points represent the mean and standard deviation (*n* ≥ 3). *Abbreviations*: PI, prophase-I; MI, metaphase-I; MII, metaphase-II; hpf, hours post-fertilization; b-isox, biotinylated-isoxazole; λ-PPase, lambda phosphatase; In, input; Prog. or P, progesterone; DE, phosphomimetic mutant; A, phosphonull mutant; NTD, N-terminal domain; NL, no-lysate; NI, not-injected

We first sought to identify a biological model in which all four vertebrate CPEBs are expressed and regulated (i.e., in their switch from a repressor to an activator). CPEB1 and CPEB4 have been extensively studied in meiotic cell cycle progression (in *Xenopus laevis* oocytes), where they are both expressed and sequentially regulated [7, 13]; therefore, we analyzed if CPEB2 and CPEB3 are also expressed in *Xenopus* oocytes. To enrich RNA-binding proteins that contain low-complexity regions, we performed biotinylated isoxazole (b-isox) precipitation [23], followed by electrophoresis and immunoblotting (Fig. 1A) or tandem mass spectrometry (MS/MS) (Fig. 1B), of oocyte lysates at three maturation stages: prophase I (PI) arrest, metaphase I (MI) entry, and metaphase II (MII) arrest. All four CPEBs were detected by MS/MS, with fewer unique peptides of CPEB2–4. The high sequence identity within the CPEB2–4 subfamily lowers the number of potential unique peptides. Nonetheless, it would seem, in agreement with high throughput proteomics studies [24, 25], that CPEB1 expression in oocytes is higher than for the CPEB2–4 subfamily. Immunoblotting of CPEB2 and CPEB3 showed that they (i) were present at PI, (ii) shifted to slower mobility after meiotic resumption, and (iii) decreased in levels in MII and in embryonic mitosis. This pattern of expression is similar to that of CPEB1 [26] and complementary to that of CPEB4 [13], despite having a much lower abundance of protein. To determine if the observed shifts were caused by phosphorylation, we expressed HA-tagged CPEB2 and CPEB3 and induced cell cycle progression with progesterone. Both tagged proteins exhibited mobility changes similar to their endogenous counterparts, with concomitantly decreased protein levels for HA-CPEB2 (Fig. 1C, D). The mobility changes were partially or totally abrogated by phosphatase treatment for HA-CPEB2 and HA-CPEB3 (Fig. 1E).

We next aimed to identify the phosphorylated residues in the CPEBs. For CPEB3, we expressed the HA-tagged protein and then collected samples in PI arrest or after progesterone-induced meiotic resumption (+ prog). MS/MS analysis of excised SDS-PAGE bands showed a global increase in HA-CPEB3 phosphorylation in metaphase and identified a relative gain of phosphorylation at 11 sites, 10 of which are in consensus proline-directed motifs (Fig. 1F). Furthermore, all 11 of the sites were in the NTD of CPEB3 and included the majority of the predicted proline-directed motifs (Fig. 1G). Mutation of these 11 residues abolished the in vitro phosphorylation of recombinant CPEB3-NTD using extracts from cell cycle-synchronized oocytes as the source of kinases, indicating that all key residues were identified (Fig. 1H, Additional file 1: Fig. S2C). Indeed, when expressed in oocytes, a non-phosphorylatable CPEB3 mutant failed to display the mobility shift in MI, whereas a phosphomimetic mutant in PI arrest displayed the same mobility as the wild-type (WT) in MI (Fig. 1I). Because CPEB2 was destabilized concomitantly with phosphorylation, it was not possible to perform MS/MS. However, as both CPEB3 and CPEB4 were phosphorylated at proline-directed sites, whose number but not the position is relevant for its function [7], and with the same kinetics as CPEB2 (Additional file 1: Fig. S2B, D), we directly tested if that was also the case for CPEB2 by mutating the 20 predicted proline-directed sites to A or D/E (Fig. 1J). Mutation of these residues greatly reduced (but did not abolish) in vitro phosphorylation of recombinant CPEB2-NTD using extracts from cell cycle-synchronized oocytes as the source of kinases (Fig. 1K, Additional file 1: Fig. S2B). When expressed in oocytes, a non-phosphorylatable CPEB2 mutant failed to display the mobility shift in MI, whereas a phosphomimetic mutant in PI-arrest displayed the same mobility as the WT in MI (Fig. 1L).

Because the phosphorylation events were mainly at proline-directed sites and by kinases activated in MI and MII, we next tested the inhibitors of the main proline-directed M-kinases: Cdk1 (RO-3306), ERK (FR180204), and MEK (U0126). We observed a dose-dependent inhibition of phosphorylation of CPEB2 and CPEB3 with Cdk1 and ERK inhibitors, but not with MEK inhibitors (Fig. 1M, Additional file 1: Fig. S2E).

Altogether, we found that CPEB2 and CPEB3 followed a similar expression pattern as CPEB1 but were subjected to the same regulation via phosphorylation as CPEB4. We did not detect sumoylation or ubiquitination in meiotic oocytes for either CPEB2 or CPEB3 (Additional file 1: Fig. S3).

### CPEB1 forms mRNP foci while CPEB2–4 assemble liquid-like condensates, also in the absence of RNA binding

**Fig. 2:**
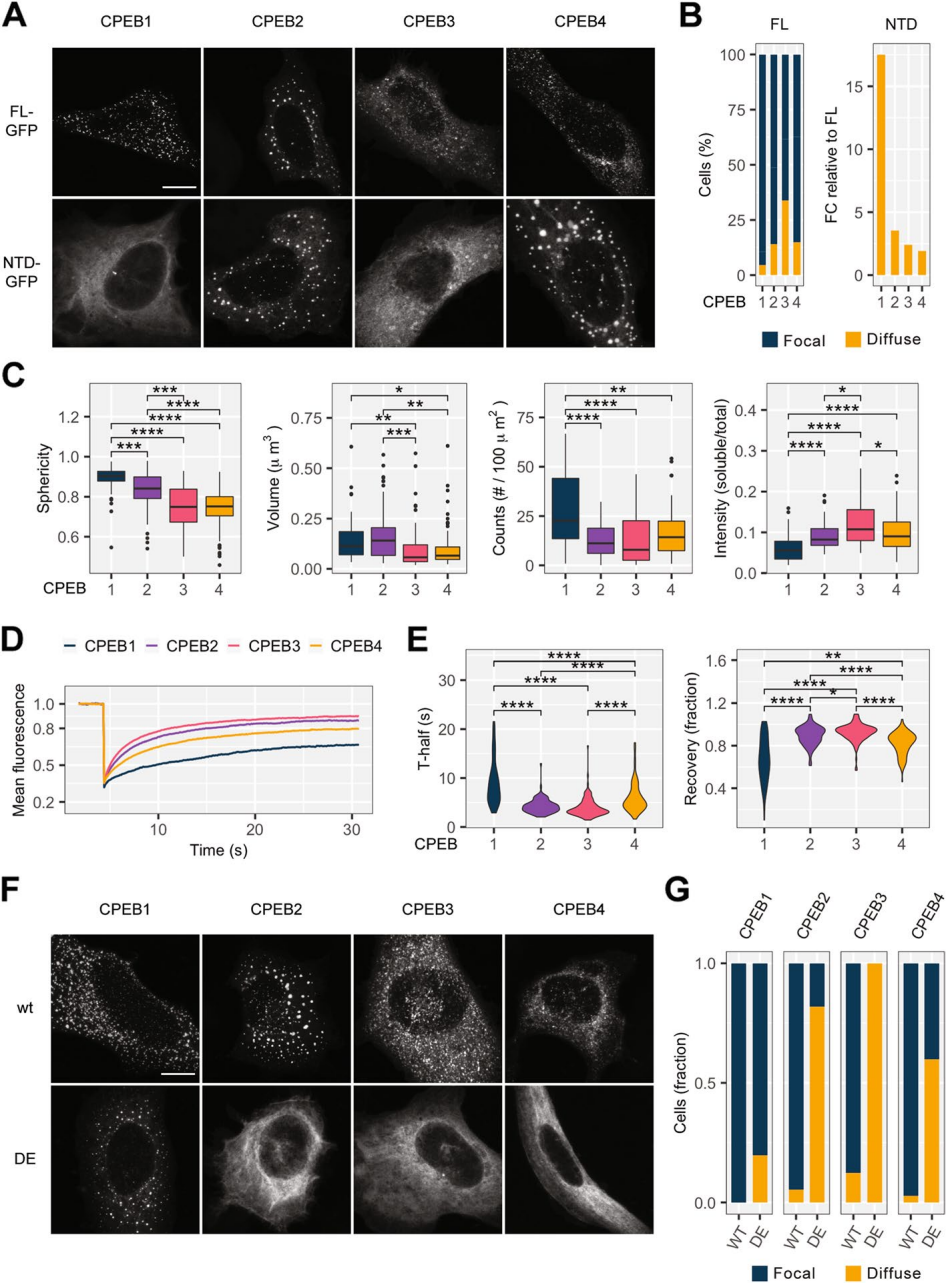
The four CPEBs form cytoplasmic foci that possess distinct biophysical properties. **A** U-2 OS cells overexpressing either full-length or NTD CPEB1-4-GFP fusions. Scale bar, 10 μm. **B** Left: percentage of cells displaying “focal,” in blue, versus “diffuse,” in orange, cytoplasmic distribution of full length (FL) CPEB1-4 (1, 2, 3, 4), the latter defined by the absence of foci. Right: fold change (FC) increase in the number of “diffuse” cells in the N-terminal domain (NTD) constructs relative to full length (FL). CPEB1, *n* = 67; CPEB2, *n* = 72; CPEB3, *n* = 65; CPEB4, *n* = 67; CPEB1-NTD, *n* = 65; CPEB2-NTD, *n* = 65; CPEB3-NTD, *n* = 59; CPEB4-NTD, *n* = 63. **C** Quantification of CPEB1-4-GFP cytoplasmic foci features: sphericity, volume and number, and ratio soluble-to-total fluorescence intensity. *n* as specified for **B**. **D** The mean fluorescence recovery upon photobleaching (FRAP) curves of CPEB1-4-GFP. **E** Distribution of the half-time of recovery (t-half) and recovery fraction parameters obtained from the FRAP curves. CPEB1, *n* = 82; CPEB2, *n* = 95; CPEB3, *n* = 106; CPEB4, *n* = 109. **F** U-2 OS cells overexpressing either *wt* or phosphomimetic CPEB1-4-GFP fusions. Scale bar, 10 μm. **G** Fraction of cells displaying “focal,” in blue, versus “diffuse,” in orange, cytoplasmic distribution in *wt* versus phosphomimetic mutants. CPEB1 wt, *n* = 72; CPEB1-DE (6D-DD), *n* = 71; CPEB2 wt, *n* = 74; CPEB2-DE (20 DE), *n* = 83; CPEB3 wt, *n* = 65; CPEB3-DE (18 DE), *n* = 68; CPEB4 wt, *n* = 73; CPEB4-DE (12 DE), *n* = 72. In **C** and **E**, comparisons between the groups were carried out using the Kruskal–Wallis test (significance level 5%) and post hoc Dunn’s test with Holm’s correction. Significance scale: ****P-adj < 0.0001; ***P-adj < 0.001; **P-adj < 0.01; *P-adj < 0.05, non-significant differences not indicated. *Abbreviations*: FL, full-length; NTD: N-terminal domain; wt, wild-type; DE, phosphomimetic mutant

To test if all four CPEBs are equally capable of forming large assemblies or even phase-separated condensates (like CPEB4), we expressed GFP-tagged *Xenopus* CPEB1–4, either as full length (FL) or mutants lacking the RNA-binding domains (NTDs) [5, 16], in U-2 OS cells (Fig. 2A, B). All four CPEBs formed foci; however, CPEB3 was the least efficient and displayed a mix of focal and soluble distribution. In the absence of RNA-binding domains, the CPEB1 distribution completely changed to a diffuse pattern. In contrast, the NTDs of CPEB2 and CPEB4 were still able to form assemblies, whereas CPEB3 displayed an intermediate behavior with a higher tendency to form irregularly shaped foci in the cytoplasm. Next, we measured the morphological parameters of FL-CPEB foci (Fig. 2C) and found that CPEB1 assemblies were rounder and occurred at greater density, while CPEB3 had a stronger tendency towards diffused patterns, as compared to assemblies of the other CPEBs. To better characterize the nature of these assemblies, we performed live imaging and fluorescence recovery after photobleaching (FRAP) experiments determining the half-time of maximum recovery (T-half) and fraction of recovery (Fig. 2D, E). Here, a fast recovery rate indicates that the molecules forming the granules have a liquid-like behavior and are in constant exchange with the surrounding cytoplasm. CPEB1 had the slowest and least efficient recovery rate, indicating that it had a less dynamic, less liquid-like behavior than the other CPEBs. Furthermore, consistent with the liquid–liquid phase separation of CPEB1, we observed fusion events over time as well as dissolution upon treatment with 1,6-hexanediol (Additional file 1: Fig. S4A, B). Indeed, CPEB1 granules tended to move across wide areas within the cytoplasm; the smaller particles (below percentile 15%) displayed fast and geometrically straight displacement events characteristic of cytoskeleton-associated mRNPs (Additional file 1: Fig. S4C). In contrast, each condensate with CPEB2, CPEB3, or CPEB4 displayed a Brownian movement with little-to-no positional displacement. Finally, we tested whether the transition between the liquid-like droplet state and the diffuse state of CPEBs was regulated by phosphorylation events by overexpressing WT or phosphomimetic CPEB1–4-GFP (Fig. 2F, G). For CPEB1, the phosphomimetic mutant did not change the distribution of its aggregates. In contrast, the CPEB2–4 phosphomimetic mutants displayed a diffuse pattern.

Altogether, these results indicate that the properties of CPEB1 assemblies are more similar to ribonucleoprotein complexes (mRNPs), which require RNA binding for their formation. CPEB2–4, however, undergo liquid–liquid phase separation. These liquid-like droplets do not require RNA for their formation.

### CPEB1 is in the vicinity of numerous mRNP components, including CCR4-NOT

**Fig. 3:**
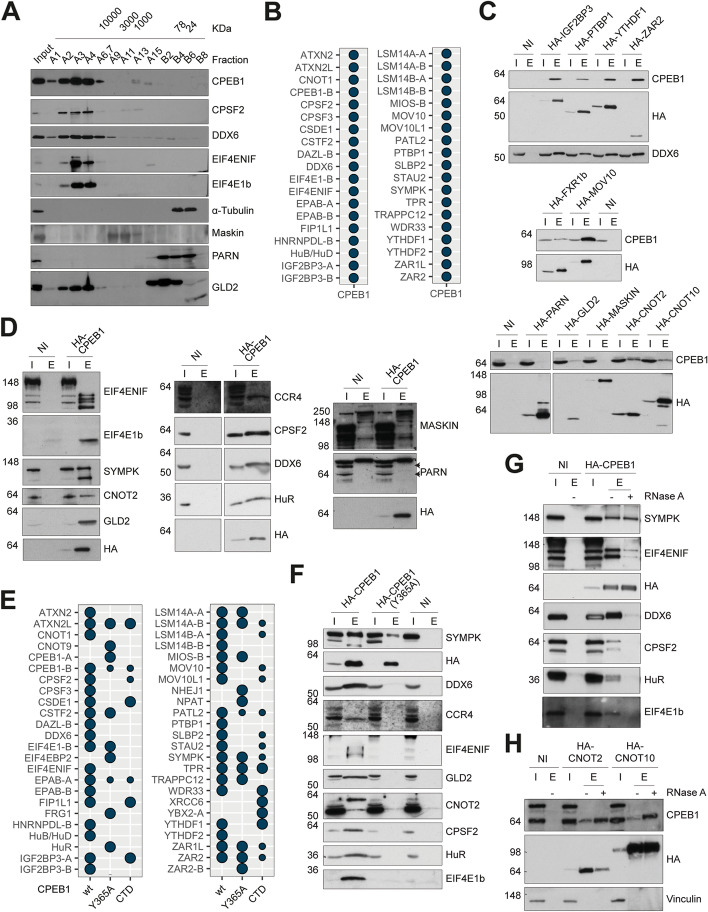
CPEB1 complex composition. **A** Western blots against the indicated proteins from several fractions of a gel-filtration fast protein liquid chromatography (GF-HPLC) done in PI-oocyte extracts. Approximate size of the fractions is noted above in KDa. **B** CPEB1 proximome in *X. laevis* PI oocytes, determined by BioID (*n* = 4). Hits include proteins enriched in CPEB1-BirA and BirA-CPEB1, relative to BirA alone. Hits meet the following criteria: either they have a positive fold change and *P*-adj < 0.05 relative to BirA alone or have 3 to 4 missing values in the control against 1 or none in the condition and a high relative abundance (greater than 25th percentile). **C** Co-immunoprecipitation of endogenous CPEB1 with the indicated HA-tagged baits (*n* = 2). **D** Co-immunoprecipitation of endogenous preys with HA-tagged CPEB1 (*n* = 3). The bands corresponding to PARN are indicated with arrows. **E** CPEB1, CPEB1(Y365A), and CPEB1-CTD proximomes determined by BioID in *X. laevis* PI oocytes (*n* = 4). The hits include the N-terminal and C-terminal BirA fusions and are defined as described in **B**. The size of the dots indicates the significance of the enrichment. **F** Co-immunoprecipitation of endogenous preys with HA-tagged CPEB1 and CPEB1(Y365A) (*n* = 2). **G** Co-immunoprecipitation of endogenous preys with HA-CPEB1 upon RNase A treatment (*n* = 3). **H** Co-immunoprecipitation of endogenous CPEB1 with the indicated HA-tagged baits upon RNase A treatment (*n* = 2). *Abbreviations*: I, input; E, elution; NI, not-injected; CTD, C-terminal domain. Indicated molecular weights are expressed in KDa

To identify the composition of the CPEB assemblies, we first purified the endogenous CPEB1-containing complexes using size fractionation of PI-arrested *Xenopus* oocyte cytoplasmic extracts. CPEB1 is the most abundant CPEB in these extracts, which corresponded to the translational repressor complex; while this complex has been previously studied, the results were discordant, leading to several mutually incompatible models for CPEB1-mediated translational repression [4]. After size fractionation, we performed immunoblot of alternating fractions ranging in molecular weights from > 10,000 KDa to < 24 KDa. In these fractions, we detected CPEB1 and proteins that have been found associated with CPEB1-repressor complexes [4]. Specifically, CPEB1 co-fractionated with CPSF2, DDX6, eIF4ENIF, and eIF4E1b, but not with Maskin nor PARN (Fig. 3A) that are proteins previously described to be involved in CPEB1-mediated repression [27, 28]. Of note, the poly(A) polymerase GLD2, which has been implicated in CPEB1-mediated cytoplasmic polyadenylation [29], was resolved as a doublet in *Xenopus* oocyte extracts, and only the fast-migrating band co-fractionated with CPEB1. Overexpressed HA-GLD2 co-migrated with the slow-migrating band. Thus, it was unclear if the protein co-fractionated with CPEB1 was a modified form, a splice variant or an unrelated cross-reacting band (of note, MS/MS analysis of the fraction containing CPEB1 did not detect GLD2).

Next, we sought to identify the CPEB1 proximity interactors (from now on proximome) by adapting the BioID methodology [30] to *Xenopus* oocytes; for this, we expressed CPEB1 fused to the BirA enzyme either at the N- or C-terminus. BirA was also expressed alone as a control. We defined a total of 30 high-confidence proximome interactors in the CPEB1 proximome (Fig. 3B). These proteins included previously reported CPEB1-associated proteins, such as CPSF2, DDX6, Symplekin (SYMPK), ePAB, eIF4ENIF, eIF4E1b, and endogenous CPEB1 (reviewed in [4]). Neither PARN nor Maskin were detected. We also identified novel CPEB1 proximome components, such as YTHDF1, ATXN2, SLBP2, MOV10, PTBP1, IGF2BP3, and CNOT1. We next validated some of these proximity interactions by co-immunoprecipitation (Fig. 3C, D). HA-CPEB1 efficiently co-immunoprecipitated HuR, DDX6, eIF4ENIF, eIF4E-1b, CNOT2, CPSF2, and SYMPK. Components of the CCR4-NOT complex, including the catalytical subunit CCR4, CNOT2, and CNOT10, also co-immunoprecipitated with CPEB1. The complex included m^6^A readers, such as YTHDF1 and IGF2BP3, as well as other RNA-binding proteins implicated in translational repression, such as ZAR2, PTBP1, MOV10, and FXR1b. However, neither Maskin nor PARN co-immunoprecipitated with CPEB1 while GLD2 did co-immunoprecipitate. As the assembly of repressive CPEB1 aggregates required mRNA binding, we overexpressed a mutant that had a reduced RNA binding capacity (CPEB1(Y365A)) and another one that lacked the RNA-binding region altogether (thus, CPEB1 C-terminal domain (CTD)) [5, 16]. BioID showed a reduction or loss of CPEB1 interactors with both mutants (Fig. 3E). CPEB1 co-immunoprecipitation confirmed that RNA binding was required for interactions of DDX6, CCR4, CNOT2, eIF4ENIF, eIF4E-1b, CPSF2, and HuR with CPEB1, while no RNA binding was required for CPEB1 and SYMPK interaction (Fig. 3F). Treatment of the lysates with RNase prior to immunoprecipitation further confirmed the RNA dependency of said interactions (Fig. 3G, H).

**Fig. 4:**
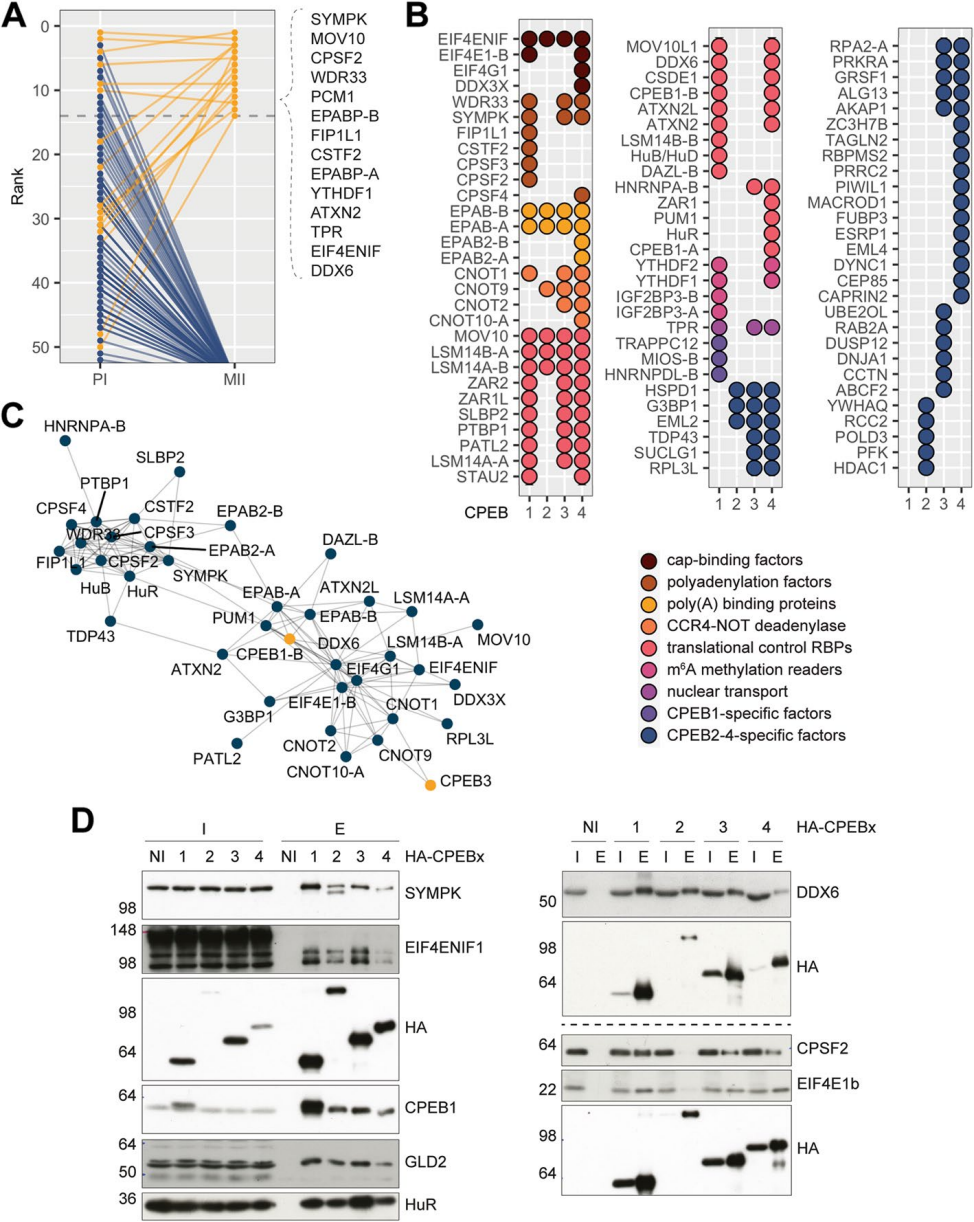
Comparative CPEB1-4 complex composition. **A** BioID-detected changes in the CPEB1(6A) proximome between PI (blue) and MII (yellow) (*n* = 3). The preys are compared in terms of their enrichment rank. Note that these ranks admit draws. **B** CPEB1-4 proximomes in *X. laevis* PI oocytes, determined by BioID (*n* = 4). Hits include proteins enriched in N-terminal and C-terminal BirA fusions, relative to BirA alone. Hits meet the following criteria: they either have a positive fold change and *P*-adj < 0.05 relative to BirA alone or have 3 to 4 missing values in the control against 1 or none in the condition and a high relative abundance (greater than 25.th percentile). **C** STRING network plot of the CPEB proximome in *Xenopodinae* displaying high confidence interactions (score > 0.7) and only networks with more than one interaction. CPEBs have been highlighted in yellow. **D** Co-immunoprecipitation of endogenous preys with HA-tagged CPEBs confirmed by Western blot using specific antibodies (*n* = 3)

Finally, to identify which of these proteins were specific to the CPEB1-assembled repressor complex, we performed CPEB1-BioID in oocytes treated with progesterone (Fig. 4A), which promotes the CPEB1 switch from repression to activation (reviewed in [4]). Comparative enrichment of proteins in both conditions (i.e., with or without progesterone) showed that several proteins were no longer present in the proximome of the activatory CPEB1 complex (including the CCR4-NOT complex, HuR/PTBP1, and LSM14/PATL2), while others remained present (including Symplekin, CPSF2, eIF4ENIF, DDX6, and ePABP). The latter group could be “pre-loaded” [31] components of the CPEB1 activatory complex [32, 33, 34]. Alternatively, they could be proteins generically associated with mRNPs regardless of their fate, whether nucleocytoplasmic transport, cytoplasmic localization, repression, or activation; in this case, these proteins are most likely not the direct mediators of translational repression by CPEB1.

### CPEB1 and CPEB2–4 have distinct proximomes but share CCR4-NOT components involved in repression

Because of the differential behavior in condensate dynamics and regulation by phosphorylation, we next analyzed the composition of the complexes assembled by the four CPEBs by identifying their respective proximomes (including both N- and C-BirA fusions) in translation repression conditions (Fig. 4B, Additional file 1: Fig. S5). We expanded prior knowledge of CPEB4 proximity interactors [35] to a new cellular context, where the CPEBs exert translational repression, in addition to comparing to the other members of the protein family. Of note, due to its lower stability, overall labeling for the CPEB2 fusions was lower than for the other CPEB fusion proteins, whereas CPEB4 and CPEB1 yielded the most efficient labeling. To overcome this limitation, in the visualization (Fig. 4B), we grouped the identified proteins in functional categories, rather than comparing individual proteins. Indeed, we found that CPEB1–4 share a core of proximome components: all four CPEBs labeled cap-binding proteins, components of the polyadenylation machinery, PABP, components of the CCR4-NOT complex, and some of the previously identified RNA-binding proteins (such as MOV10). Like CPEB1, the CPEB4 proximome also included m^6^A readers. In addition, those of CPEB2–4 included a new set of proximal proteins not identified for that of CPEB1. We also found proximity interactors specific to individual CPEBs. Some of the CPEB3/CPEB4-specific preys have been implicated in the regulation of mRNA stability, localization, and translation, as is the case of G3BP1 [35], PRKRA [36], DDX3X [37], ESRP1 [38], and CAPRIN2 [39]. When the entire CPEB1–4 proximome was analyzed in STRING [40], it formed a network of highly interconnected proteins organized in two nodes (Fig. 4C): the first node included polyadenylation factors such as CPSF proteins, CSTF2, and Symplekin, and the second included the deadenylation complex CCR4-NOT as well as the cap-associated proteins eIF4E1-b, eIF4G, and eIF4ENIF. Given the size of the CPEB1-mRNP (Fig. 3A), it would be plausible that these two nodes integrated into the same mRNPs.

In the CPEB proximomes, we also identified endogenous CPEB1, which suggested the possibility of co-localization of different CPEBs in the same condensates. To test this possibility, we first performed co-immunoprecipitation of all four HA-tagged CPEBs and analyzed their associations with endogenous CPEB1; these results showed that indeed all CPEBs were part of the same complexes (Fig. 4D). Furthermore, by co-expressing CPEB1-mCherry with GFP fusions for all 4 individual CPEBs, we observed a strong colocalization among CPEBs, as well as to CNOT2, another proximome component (Additional file 1: Fig. S6).

Thus, all four CPEB repressive complexes share the interaction with the CCR4-NOT complex but also have CPEB-specific co-factors. Nevertheless, at a larger scale, they appear to co-localize in the same subcellular structures, even if with different regulations and physical properties. Accordingly, superresolution/ultraexpansion microscopy (U-ExM) showed an intradroplet segregation between different CPEBs (Additional file 1: Fig. S7A), while co-FRAP showed different exchange dynamics between the soluble and aggregated fractions (Additional file 1: Fig. S7B).

### CPEB1 and CPEB2–4 share target mRNAs but have different motif preferences

**Fig. 5:**
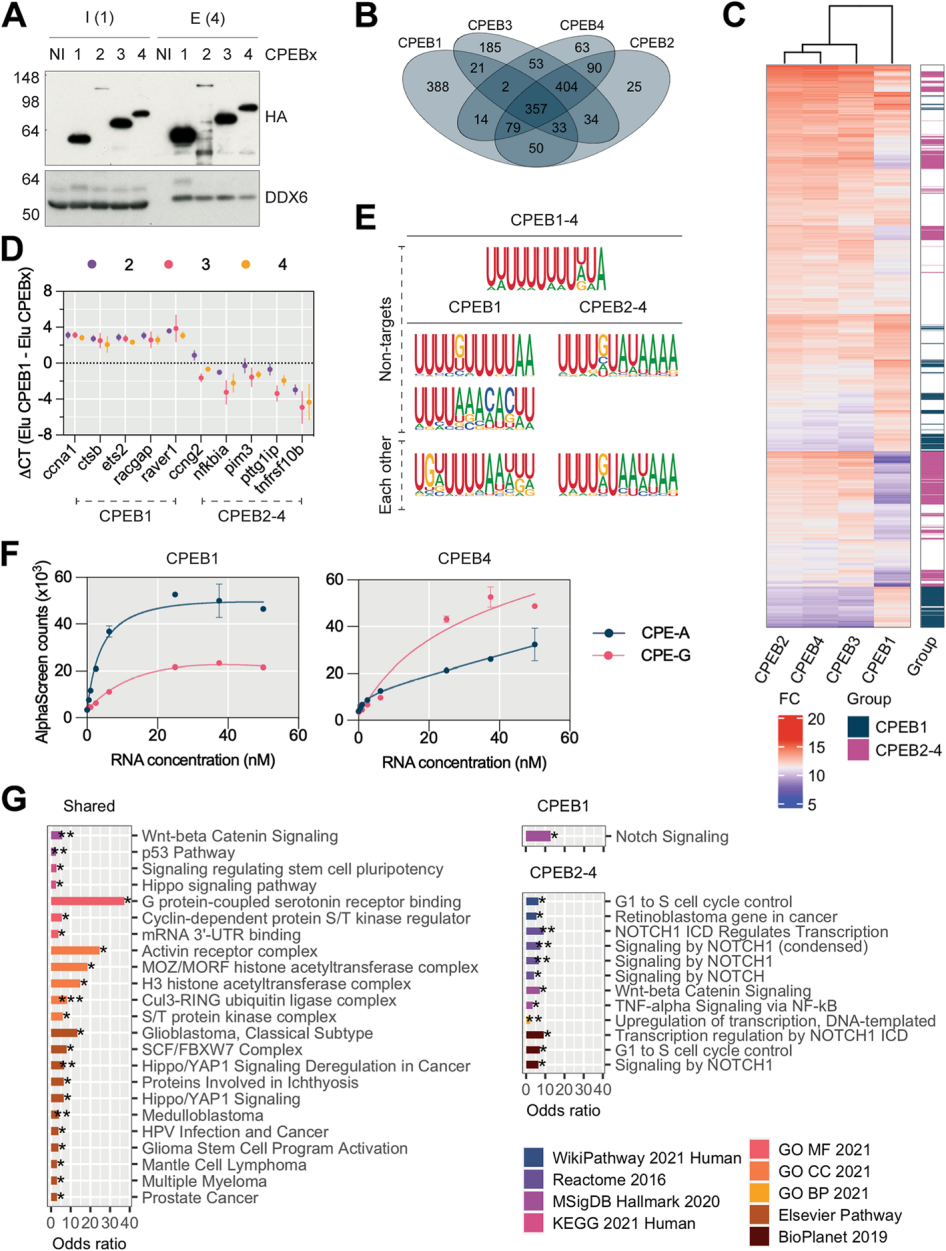
CPEB1 and the CPEB2-4 subfamily target distinct mRNA subsets. **A** Western blot detection of HA and DDX6 of HA immunoprecipitates from HA-CPEB1-4 overexpressing oocytes (*n* = 3). The number of oocytes loaded is indicated in parentheses. I, input; E, eluate; NI: not-injected. **B** Overlap between the CPEB1-4 targets defined from RIP-Seq experiments. Targets are at least fourfold enriched relative to the input (*P*-adj ≤ 0.05) and twofold relative to the not-injected background control IP (*P*-adj ≤ 0.05). **C** Left: CPEB-mRNA enrichment heatmap for targets of at least one CPEB. The enrichment is expressed as the module of the two centered fold changes. The clustering tree was created with the full-linkage method. Right: CPEB1-CPEB2-4 differential enrichment heatmap. Colored genes are enriched at least twofold in one group versus the other (*P*-adj ≤ 0.05). **D** RIP-qPCR enrichment (expressed as delta CT) of indicated candidates in the CPEB1 IP relative to CPEB2/3/4 IPs. The candidates are either CPEB1-preferential targets or CPEB2-4-preferential targets, as indicated with dashed lines. Data points represent the mean and standard deviation (*n* = 3). **E** Motifs differentially enriched in the 3′ UTRs of targets of any CPEB-, CPEB1-, or CPEB2-4-preferential targets relative to RIP-Seq input minus targets and each group of preferential targets relative to the other, as determined with HOMER. The background used is indicated for the table rows. **F** AlphaScreen assay of purified CPEB1 and CPEB4 (50 nM) binding to CPE-A or CPE-G oligonucleotides. Error bars represent the standard deviation of the technical replicates (*n* = 2). The experiment was performed in triplicate. **G** Gene set enrichment in CPEB1-preferential targets (234 genes), CPEB2-4-preferential targets (414), or non-preferentially regulated targets (shared, 1148) determined with Enrichr. Only ontologies within “pathways” and “ontologies” with significant gene sets are included. Signaling by NOTCH1 (condensed) includes four redundant Reactome 2016 categories. GO MF, CC, and BP refer to molecular function, cellular component, and biological process, respectively. Significance scale: ****P-adj < 0.0001; ***P-adj < 0.001; **P-adj < 0.01; *P-adj < 0.05

Finally, we sought to determine the mRNA target specificity for the four CPEBs. For this, we performed RNA immunoprecipitation sequencing (RIP-Seq) for each HA-tagged CPEB in PI-arrested oocytes. To avoid differential immunoprecipitation efficiencies due to differential antibody affinities, individual HA-CPEBs were expressed in oocytes and immunoprecipitated with an HA antibody (Fig. 5A, Additional file 1: Fig. S8A). For each immunoprecipitated CPEB, we defined a high-confidence target subset, of approximately 1000 mRNAs (Additional file 1: Fig. S8B-D). A total of 1798 mRNAs were associated with at least one CPEB, with 357 transcripts being bound by all four CPEBs (Fig. 5B). CPEB2–4 shared the majority of their targets (761), while CPEB1 had 388 unique hits and was therefore the most dissimilar (Fig. 5B). Unsupervised clustering of the CPEB1–4 targetomes revealed that CPEB1 and CPEB2–4 formed two well-differentiated groups (Fig. 5C); hence, we defined two subsets of CPEB1 and CPEB2–4-preferential targets by differential enrichment analysis, some of which were validated by RIP-qPCR (Fig. 5D, Additional file 1: Fig. S8H). To determine if these differences originated from differential *cis*-acting motifs, we performed a differential de novo motif enrichment analysis of CPEB1 versus CPEB2–4 target subsets (Fig. 5E and Additional file 2: Table S1). Targets of any CPEB had longer 3′ UTRs than non-target mRNAs (median of 1489 nucleotides versus 531; Additional file 1: Fig. S8F) were enriched in U-rich motifs (Fig. 5E and Additional file 2: Table S1) and in the motif architectures previously described for CPEB1 targets (Additional file 1: Fig. S8G) [2]. Furthermore, CPEB1-preferential targets were enriched in (i) a U-extended “UUUUAA” motif, as compared to the non-CPEB-bound mRNAs and (ii) the canonical “UUUUAAU” CPE, as compared to the CPEB2–4-enriched targets (Fig. 5E and Additional file 2: Table S1). The reciprocal comparison showed that preferential targets of CPEB2–4 were enriched in a “UUUUGUA” motif, both relative to the input and relative to CPEB1-preferential targets (Fig. 5E and Additional file 2: Table S1). The affinity of CPEB1 and CPEB2–4 for the canonical CPE (CPE-A) or a CPE with a G substitution (CPE-G), respectively, was validated with an AlphaScreen binding assay (Fig. 5F).

In order to find the functional differences between the genes regulated by one or the other subfamily, we performed gene set enrichment analyses with Enrichr [41], for common vs CPEB2–4- or CPEB1-specific targetomes (Fig. 5G). We did not find any functional categories uniquely enriched for CPEB1. On the other hand, G1-S cell cycle control and TNF-alpha signaling were uniquely enriched among CPEB2–4 targets. Common targets to all four CPEBs included several elements related to cell fate, such as pluripotency, cell division/arrest, and cancer.

## Discussion

Collectively, analyses of CPEB complexes show that all four members of the family share three main properties. First, all four can recruit the CCR4-NOT deadenylase to the cytoplasmic CPEB-repressor complexes (consistent with the findings by Poetz et al. for the CPEB4 manuscript submitted to Genome Biol). Thus, while PARN could be implicated in nuclear CPEB-mediated deadenylation [27, 42], maintenance of a short poly(A) tail in the cytoplasm is probably mediated by CCR4-NOT. Furthermore, all four CPEBs interact with other RNA-binding proteins that are not specific for the repression/deadenylation complex but can be “pre-loaded” [31] components of the CPEB1 activatory complex [32, 33, 34] or proteins generically associated with mRNPs. Second, all four CPEBs recognize the “canonical” CPE (UUUUA_(1–2)_U); we found that only a fraction of the CPEB-regulated transcripts are shared by all four CPEBs. This observation is consistent with the structure of the CPEB1 and CPEB2–4 RRMs [5]. Third, all four CPEBs can co-localize in the same condensates with liquid-like properties (LLDs).

However, and consistently with their evolutionary distances, we found that CPEBs can be classified into two different groups or subfamilies, one including CPEB1 and the othe1r including CPEB2–4. These two groups have specific properties in target/motif recognition, large-order complex co-factors, and dynamic properties and regulation during cell cycle. Thus, while CPEB1 only recognizes canonical CPEs, CPEB2–4 also bind to “G-variants” (UUUUGU), reminiscent of the Orb/Orb2 targets [16] and consistent with the findings by Poetz et al. for CPEB4 [43]. This differential binding implies that, while CPEB1 targets are shared with CPEB2–4, this second CPEB subfamily has also other specific targets. CPEB2–4-specific targets are enriched in the G1/S cell cycle and TNF-alpha.

The larger order complexes assembled by CPEB1 exhibited properties more closely related to canonical RNP complexes with respect to their stability, composition, and association with cytoskeleton and motors; this is consistent with CPEB1’s association to dynein and kinesin [44]. These complexes required mRNA binding for their assembly and localization to the same LLDs. Even once in the condensates, their exchange with the soluble form was much slower than for the other CPEBs. On the other hand, condensates assembled by CPEB2–4 showed the properties of canonical LLDs in terms of size, morphology, and dynamic properties. Unlike CPEB1, the intrinsically disordered regions of CPEB2–4 were able to assemble LLDs in the absence of RNA binding. These properties are consistent with the distribution of unstructured regions in the N-terminal part of CPEBs, the charge distribution, and the presence of regions with “prion properties.” When co-expressed, CPEBs localized to the same larger-order aggregates; however, they maintained their individual properties, suggesting heterogeneity or even spatial sub-compartmentalization within the LLD. This is reflected in their respective proximomes, which include both common and CPEB-specific cofactors.

Regulation by phosphorylation also defines two subfamilies. Thus, CPEB1 was activated by a single phosphorylation (by AurkA), which remodels the composition of the CPEB1-assembled mRNP [32, 33] but not its inclusion in LLDs. Subsequent multiple proline-directed phosphorylation events promoted CPEB1 degradation. On the other hand, CPEB2–4 were activated by multiple proline-directed phosphorylation events (by ERK and Cdk1), which promoted the dissolution of the LLDs.

Therefore, the two CPEB subfamilies are regulated through distinct signaling pathways and mechanisms. It seems very unlikely that both subfamilies could be activated simultaneously. Indeed, AurkA is specifically activated at pro-metaphase and upon synaptic stimulation, while CDK1/ERK are activated starting from anaphase during the cell cycle and ERK as part of the stress responses. Accordingly, CPEB1 activation is essential in the G2/M transition, while CPEB 2–4 act later in the cell cycle [14, 15, 45]. This implies that, for cells that co-express the two subfamilies as well as for mRNAs with canonical CPEs, there will be a competitive equilibrium between a CPEB acting as a repressor and other CPEBs acting as activators. mRNAs with non-canonical G/CPEs would not be subjected to these opposing actions. In addition, regulation by a large number of phosphorylation sites in CPEB2–4, versus a single one in CPEB1, would allow for an ultrasensitive response in the CPEB2–4 subfamily [46].

Within the CPEB2–4 subfamily, there are more subtle differences in the size, shape, and dynamic behavior of the assembled LLDs with some specific co-factors recruited. However, the specificity of their functions appears to be more related to their expression patterns. For example, CPEB4 is the only member of the family expressed upon ER stress [20], and CPEB2 is regulated as part of the estrogen hormone response [15].

## Conclusions

Collectively, our comparative study of CPEB repressor complexes, regulation, and targets defined a regulatory network that potentially targets 30% of the genome, which allows fine-tuning of the translational activation in a coordinated temporal and spatial manner. Thus, it appears that the first evolutive CPEB duplication event generated two distinct but coordinated mechanisms of regulation, whereas subsequent duplications fine-tuned the response of the second subfamily.

## Methods

### Plasmids

All plasmids used for this work are listed in Additional file 2: Table S2.

### Antibodies

Antibodies used for this work are listed in Additional file 2: Table S3.

### *Xenopus laevis* oocyte preparation

Stage VI oocytes were obtained from female *Xenopus laevis* frogs and kept in Modified Bath Saline media as described previously [47]. For the overexpression experiments, oocytes were microinjected with 46 nL of 50 ng/μL in vitro transcribed RNAs using a Nanoject II microinjector (Drummond). When indicated, maturation of oocytes was induced with 10 μM progesterone (Sigma) in MBS at 18 °C or room temperature (RT). Metaphase-I was scored by the appearance of a white maturation spot (wms). Oocytes were considered to have reached metaphase-II 3 h after wms formation.

### Protein immunoprecipitation (IP)

Polyadenylated RNA (0.036 pmol) encoding for HA-protein of interest was injected into stage VI oocytes, and 16-h post microinjection oocytes were lysed in 9 μL/oocyte cold IP lysis buffer (20 mM Tris–HCl pH 8, 100 mM NaCl, 0.4% NP40, 1 mM EDTA, 1 mM MgCl_2_, 1 × Complete EDTA-free protease inhibitors (Roche)) and clarified by centrifugation. 1 × H1K phosphatase inhibitors (80 mM sodium β-glycerophosphate, 0.5 mM sodium orthovanadate) were added to the recovered aqueous phase. In IPs with RNase treatment, the lysates were incubated for 15 min at 37 °C with 0.1 μg/μL of RNase A, DNase, and protease-free (Thermo Fisher Scientific). One hundred fifty microliters of HA-conjugated magnetic beads (Pierce Anti-HA Magnetic Beads, Thermo Fisher Scientific) was used per mL of clarified lysate. Lysate and beads were then incubated for 2–20 h. The beads were washed thrice in one volume of lysis buffer. Purified proteins were eluted from the beads with Laemmli sample buffer without DTT. Supernatants were then recovered with a magnetic rack (Thermo Fisher Scientific), and beads were discarded and DTT was added to the supernantant at 180 mM final concentration. The eluates were then analyzed by Western blot (WB).

### Biotinylated-isoxazole (b-isox) precipitation

Oocytes were lysed as previously described and subjected to a second clarification. Clarified lysates were treated with 100 μM b-isox in DMSO and incubated overnight on a rotating wheel at 4 °C. Low-complexity-region-containing proteins were precipitated by centrifugation for 10 min, 10,000* g* at 4 °C. Pellets were washed twice in cold lysis buffer and resuspended in Laemmli sample buffer.

### Recombinant protein production

*E. coli* BL21 (DE3) competent cells were transformed with pET30a containing the proteins of interest (Accession numbers: CPEB1: Q91572, CPEB2: A0A1L8HTE0, CPEB3: A0AIL8FJ58, CPEB4: A0AILGV75). Bacterial pellets were lysed in cold lysis buffer (25 mM Tris pH8, 1 M NaCl, 5% glycerol, 5 mM MgCl_2_, 0.5% NP40, 10 mM imidazole, complete protease inhibitors (Roche), and 0.2 mg/ml PMSF) followed by sonication and centrifugation; the resulting supernatants were used for protein purification. The clarified lysates were incubated for 1 h at 4 °C with 4 mL Ni–NTA Agarose beads (Qiagen). After three 10-mL washes with lysis buffer 0.5 M NaCl, the beads were packed in columns and the proteins were eluted with two 1-mL 10-min incubations in elution buffer (lysis buffer 0.1 M NaCl, 1 M imidazole) and one overnight elution. The eluted fractions were dialyzed in dialysis buffer (25 mM Tris pH 8, 0.1 M NaCl, 5 mM MgCl_2_, 5% glycerol, 10 mM imidazole) for 2 h with gentle shaking (Spectra/Por Regenerated Cellulose Dialysis Membranes, 8000 MWCO, Spectrum Europe). Lastly, 0.01% NaN_3_, complete protease inhibitors (Roche), and 1 mM PSMF were added to the dialyzed purified proteins and stored at − 80 °C.

### CPEB3 phosphorylation site mapping by mass spectrometry

Overexpressed HA-CPEB3 was immunoprecipitated from 100 oocytes. Oocytes were lysed in 9 μL/oocyte of 1 × H1K buffer with 0.4% NP40, and after clarification, 1 μL of 10 × IP lysis buffer was added per 9 μL of the recovered aqueous phase. HA-conjugated beads were incubated with the oocyte lysates for 90 min, washed, and eluted with Laemmli sample buffer. Eppendorf LoBind microcentrifuge tubes (Thermo Fisher Scientific) were used throughout the protocol. The IP elutions were run on precast 7.5% gels (Mini-Protean TGX, Bio-Rad) and silver-stained using the Pierce Silver Stain for MS kit (Thermo Fisher Scientific) following the manufacturer’s instructions. The bands of interest were cut, washed with 50 mM ammonium bicarbonate and acetonitrile, and then incubated in 10 mM DTT and 50 mM iodoacetamide. Samples were digested either with trypsin (Sequencing Grade Modified Trypsin, Promega) or with chymotrypsin (V1062, Chymotrypsin Sequencing Grade, Promega). Digestions were stopped with 5% formic acid. The samples were then evaporated and reconstituted in 3% acetonitrile and 1% formic acid. For the nano-LC–MS/MS, 50% of the sample volume was used. Samples were loaded to a C18 precolumn (Acclaim PepMap100, 5 µm, 100 Å, 300 µm × 5 mm, Thermo Fisher Scientific) at a flow rate of 15 μL/min using a Dionex UltiMate 3000 chromatographic system (Thermo Fisher Scientific). Peptides were separated using a C18 analytical column (NanoEase MZ HSS T3 1.8 μm, 100 Å, 75 μm × 250 cm, Waters) with a 90-min run, comprising three consecutive steps with linear gradients from 1 to 35% B in 90 min, from 35 to 50% B in 5 min, and from 50 to 85% B in 2 min, followed by isocratic elution at 85% B in 5 min and stabilization to initial conditions (where B was 0.1% formic acid in acetonitrile, and the other eluate was 0.1% formic acid in water). The column outlet was directly connected to an Advion TriVersa NanoMate (Advion) fitted on an Orbitrap Fusion Lumos Tribrid (Thermo Fisher Scientific). The mass spectrometer was operated in a data-dependent acquisition (DDA) mode. Survey MS scans were acquired in the Orbitrap with the resolution (defined at 200 m/z) set to 120,000. The lock mass was user-defined at 445.12 m/z in each Orbitrap scan. The top speed (most intense) ions per scan were fragmented and detected in the linear ion trap. The ion count target value was 400,000 for the survey scan and 50,000 for the MS/MS scan. Target ions already selected for MS/MS were dynamically excluded for 30 s. Spray voltage in the NanoMate source was set to 1.60 kV. RF Lenses were tuned to 30%. The minimal signal required to trigger MS to MS/MS switch was set to 25,000. The spectrometer was working in positive polarity mode and singly charge state precursors were rejected for fragmentation. For peptide identification, MS/MS spectra were searched against the Uniprot *Xenopus laevis* database, release 2018_10, contaminants, and user-defined proteins using both MaxQuant software v1.6.2.6a with Andromeda search engine and Proteome Discoverer v2.1 (Thermo Fisher Scientific) with Sequest HT and Amanda search engines. Searches were run against targeted and decoy database to determine the false discovery rate (FDR). Search parameters included trypsin enzyme specificity, allowing for two missed cleavage sites, oxidation in methionine, phosphorylation in serine/threonine/tyrosine, and acetylation in protein N-terminus as dynamic modifications and carbamidomethyl in cysteine as a static modification. Peptide mass tolerance was 10 ppm, the MS/MS tolerance was 0.02 Da and minimal peptide length was 7 amino acids. Peptides with a *q*-value lower than 0.1 and FDR < 1% were considered as positive identifications with a high confidence level. For the analysis, a phosphorylation ratio (*r*) for each phosphorylation site (p-site) within CPEB3 protein was computed considering three search nodes: Andromeda, Amanda, and Sequest. For each p-site, the number of position-specific phosphorylated peptide spectrum matches (PSMs) (*N*_Phos_) and the number of non-phosphorylated PSMs (*N*_NonPhos_) were counted, from which *r* was then computed as follows:

$$r = {N}_{\mathrm{Phos}}/({N}_{\mathrm{Phos}} + {N}_{\mathrm{NonPhos}})$$

In this calculation, only those p-sites with localization probability greater than 75% were considered.

### Lambda protein phosphatase assay

Microinjected oocytes were lysed at different maturation stages in 10 μL/oocyte 1 × H1K, supplemented with 0.4% NP40 and 100 mM N-ethylmaleimide (NEM, Sigma). Homogenates were cleared, and one volume of lysate was mixed with one volume of 2 × λ-PPase master mix [2 × λ-PPase buffer, 4 mM MnCl_2_, 800 U λ-PPase (NEB)] and incubated for 1 h at 30 °C. Reactions were stopped by the addition of 1/3 sample volume of Laemmli sample buffer, boiled, and subsequently analyzed by SDS-PAGE followed by WB.

### In vitro phosphorylation assays with oocyte lysates

Phosphorylation assays were performed as described previously in [7].

### Cell culture and transient transfection

U-2 OS cells were grown in Dulbecco’s modified Eagle medium (DMEM) with 10% fetal bovine serum, 1% penicillin–streptomycin, and 2 mM l-glutamine. For fixed-cell imaging, cells were plated on 6-well plates with 2 or 3 12 mm Ø poly-lysine-coated glass coverslips (0,111,550, Marienfeld Superior) whereas for live cell imaging, cells were seeded on µ-Slide 8 well ibiTreat plates (Ibidi). Cells were transfected at 70% confluence with 3 μg of DNA using Lipofectamine LTX and Plus Reagent (Thermo Fisher Scientific) following the manufacturer’s protocol.

### CPEB1-4-GFP distribution in U-2 OS cells

Twenty-four hours post-transfection, U-2 OS cells were fixed with 4% paraformaldehyde (15,710, Aname) in PBS for 10 min at RT. Next, the cells were washed with PBS and incubated with 0.5 μg/μL DAPI (Sigma) for 10 min. The coverslips were rinsed with PBS, dried, transferred to a 76 mm × 26 mm slide (Thermo Fisher Scientific), and mounted with Prolong Gold Antifade Mountant (P36934, Invitrogen). Image acquisition was performed with a Leica SP5 confocal (Leica Microsystems) microscope, and Z-series stacks were acquired at 1024 × 1024 pixels on a using the 63 × 1.4NA oil immersion objective and 3.3 zoom factor. The Argon 488 nm (set to 20%), Diode 405 nm (set to 9%), and HeNe 633 nm (set to 15%) laser lines were used with spectral detection adjusted for the emission of GFP (HyD2 detector set at 500–550 nm, 20% gain), DAPI (HyD2 detector set at 415–480 nm, 21% gain) and far-red fluorescence (HyD2 detector set at 636–700 nm, 10% gain), respectively. For each cell, 15–25 stacks were acquired with a fixed *z*-step size of 0.2 μm. The multiposition setting (Mark and Find) was used for acquisition. Image analysis was done by manually classifying cells as either aggregate or diffuse considering the GFP signal distribution in the cytoplasm. Also, for each cell, the total and mean fluorescence intensities were measured. For the analysis of the droplet features, a thresholding mask was generated using Renyi-Entropy autothreshold on the *z*-plane with higher GFP intensity. Once applied, the 3D Object Counter plugin (min = 10 units, slice = 15, threshold = 128) was used to count the number of droplets and to obtain their size. To estimate the droplet shape distribution, the analyzed particles min setting was set to 10 units. To assess the condensation in each cell, the intensity of the soluble fraction was measured in images where the respective masks were substracted to the original images.

### Fluorescence recovery after photobleaching (FRAP)

FRAP was performed with a Spinning Disk Microscope equipped with a FRAPPA module. A total of 350 images with a size of 512 × 512 pixels were taken by experiment, 50 before the bleaching and 300 after, with a typical frame rate of 11 images per second (88 ms) with an exposure time of 50 ms on an EMCCD camera. For acquisition, AOTF 488 nm laser intensity was set at 12%, for bleaching the laser intensity was 60% in two repeats with a dwell time of 40 ms each.

For the analysis, three regions of interest (ROIs) were defined per video: cell, background, and bleaching area. The mean fluorescence intensity of these three ROIs throughout the 350 frames of the video were obtained and outputted in tabular format with ImageJ. The tables were then entered to easyFRAP-web [48] where the “initial values to discard” variable was set to 20 and full-scale normalization was chosen. The curves were fitted to a single exponential, and half time of recovery (t-half) and percentage of mobile were obtained. Comparison between group distributions was done using the Kruskal–Wallis test (0.05 significance) and post hoc Dunn’s test with Bonferroni correction for multiple testing.

### Size exclusion chromatography

Stage VI oocyte lysate was gel filtered using a Superose 6 10/300 GL column (GE Healthcare) in running buffer (10 mM Hepes pH7.5, 3 mM MgCl_2_, 5% glycerol, 100 mM KCl), calibrated with the high molecular weight Gel filtration calibration kit (GE Healthcare, thyroglobulin (669 kDa), aldolase (158 kDa), ovalbumin (43 kDa)). Twenty-four fractions were collected and TCA precipitated by adding 0.002% sodium deoxycholate and 10% TCA; the precipitates were washed twice with acetone. Alternate fractions were separated by SDS-PAGE and visualized by WB.

### *Xenopus laevis* BioID

For overexpression of BirA-CPEBs, 150 oocytes were microinjected with the corresponding in vitro transcribed polyadenylated RNAs. Injected oocytes were incubated in MBS with 20 μM biotin (Merck) for 40 h at 18 °C. For activation BioIDs, oocytes microinjected with CPEB1(6A)-BirA fusion were stimulated with 10 μM progesterone (Sigma). Oocytes were lysed in 6 μL/oocyte cold BioID lysis buffer (50 mM Tris pH7.4, 100 mM NaCl, 1 mM PMSF, and complete protease inhibitors (Roche)) and clarified twice for 15 min at 16,000* g* 4 °C. Three hundred microliters of cold BioID lysis buffer was added to 200 μL of cleared extract, and the resulting 500 μL was subjected to clearing with PD MiniTrap G-25 columns (GE Healthcare). Triton-X100 and SDS were added to a final concentration of 1.6% and 0.04%, respectively, and the volume was increased to 1 mL, maintaining all concentrations. Cleared extracts were incubated with 200 μL MyOne Dynabeads Streptavidin C1 (Invitrogen) 20 h with orbital shaking at 4 °C. After incubation, beads were washed thrice with wash buffer 1 (8 M Urea, 0.25% SDS in PBS), twice with wash buffer 2 (6 M Guanidine-HCl in PBS), once with wash buffer 3 (6.4 M urea, 1 M NaCl, 0.2% SDS in PBS), thrice with wash buffer 4 (4 M urea, 1 M NaCl, 10% isopropanol, 10% ethanol, 0.2% SDS in PBS), once with wash buffer 1, once with wash buffer 5 (8 M urea and 1% SDS in PBS) and thrice with wash buffer 6 (2% SDS in PBS). The washed beads were further washed with 50 mM Tris–HCl pH 7.4 and 50 mM NH_4_HCO_3_ pH 8. For MS sample preparation, beads were resuspended in 500 μL 3 M urea, 50 mM pH 8.0, and 5 mM DTT for 1 h with orbital shaking at RT. Next, beads were incubated with 10 mM iodoacetamide for 30 min at RT in the dark, and then 5 μL of 500 mM DTT was added. Sample volumes were brought to 1.5 mL with 50 mM NH_4_HCO_3_ pH 8, and proteins were digested on-bead with 2 μg trypsin (Promega) 16 h with orbital shaking at 37 °C. Digestion was stopped by the addition of 1% formic acid. The supernatant was recovered and desalted with PolyLC tips C18. Peptides were eluted with 80% acetonitrile and 1% formic acid. Samples were diluted to 20% acetonitrile and 0.1% formic acid and loaded into strong cation exchange columns. Peptides were eluted in 5% ammonium hydroxide and 30% methanol. Finally, samples were evaporated and reconstituted in 3% acetonitrile and 1% formic acid. For the nano-LC–MS/MS, 10% of the sample volume was used.

The Nano-LC–MS/MS was performed as specified in the “CPEB3 phosphorylation site mapping by mass spectrometry” section with few modifications: peptides were separated using a C18 analytical column (Acclaim PepMap RSLC, 2 μm, 100 Å, 75 μm x 50 cm, nanoViper, Thermo Fisher Scientific) with a 120-min run; the ion count target value was 10,000 for the MS/MS scan; target ions already selected for MS/MS were dynamically excluded for 15 s and minimal signal required to trigger MS to MS/MS switch was set to 5000, and activation Q was 0.250. MS/MS spectra were searched against Uniprot *Xenopodinae* release 2017_02, contaminants, and user-defined proteins using Proteome Discoverer v2.1.0.81 (Thermo Fisher Scientific) with Sequest HT search engine. Searches were run against targeted and decoy database to determine the false discovery rate (FDR). Search parameters included trypsin enzyme specificity, allowing for two missed cleavage sites, oxidation in methionine, and acetylation in protein N-terminus as dynamic modifications. When specified, biotin in lysine was included as a dynamic modification as well. Peptide mass tolerance was 10 ppm, and the MS/MS tolerance was 0.6 Da. Peptides with a *q*-value lower than 0.1 and a FDR < 1% were considered as positive identifications with a high confidence level. Log_10_(iBAQ) values were used as protein intensity. Percentile normalization of the data was performed to minimize batch effects across biological replicates. *k*-nearest neighbors (kNN) were used for missing value imputation (function impute.knn from the impute R package [49, 50]) with *k* set to 10. Only the cases with one or two missing values were imputed. Cases with 3 or 4 missing observations were manually included only when the 3 or 4 missing occurred in the control condition and the test condition had 1 or none plus, the average log_10_(iBAQ) of the test was not within the 25% percentile of values detected in the whole sample (so as to filter out weaker-intensity proteins). Candidate interactions were found following a differential expression analysis, using functions lmFit and eBayes from the limma R package [51]. Biological replicates were used as adjusting variables in the model when needed.

### CPEB1-4 RNA-IP(RIP)-Seq

Sixty to 80 oocytes were used for RIP. Sixteen hours post-microinjection, oocytes were lysed with IP lysis buffer (20 mM Tris–HCl pH 8, 100 mM NaCl, 0.4% NP40, 1 mM EDTA, 1 mM MgCl_2_, 1 × Complete EDTA-free protease inhibitors (Roche) and clarified by centrifugation. 1 × H1K phosphatase inhibitors (80 mM sodium β-glycerophosphate, 0.5 mM sodium orthovanadate) were added to the recovered aqueous phase, and a second clarification step was performed, then the lysates were supplemented with 0.5 U/μL of Ribolock (Thermo Fisher Scientific). One hundred fifty microliters of HA-conjugated magnetic beads (Pierce Anti-HA Magnetic Beads, 88,837, Thermo Fisher Scientific) was used per 1 mL clarified lysate which were then incubated with HA-beads for 2 h with rotation at 4 °C. Beads were washed thrice with one volume of Ribolock-supplemented cold lysis buffer. One of 10 lysate-bead-slurry was used for protein extraction and 9/10 to RNA extraction. RNA was eluted by Proteinase K digestion. Briefly, the beads slurry or inputs were incubated in 400 μL of Proteinase K buffer (200 mM Tris–HCl pH 7.5, 100 mM NaCl, 10 mM EDTA, 1% SDS, 40 U/μL Ribolock) with 200 μg/mL Proteinase K (Proteinase K, recombinant, PCR grade, Roche) for 30 min at 37 °C. Subsequently, the RNA in the supernatant was purified using TRIzol Reagent (Thermo Fisher Scientific) and chloroform followed by ethanol precipitation. The pellet was reconstituted in 40 μL (inputs) or 15 μL (eluates) of TURBO DNase premix (1 × TURBO buffer, AM1907, Invitrogen, supplemented with 1 U/μL Ribolock) and treated with 1 μL TURBO DNase for 30 min at 37 °C. For library preparation and sequencing RNA samples were quantified by fluorometry with Qubit RNA HS Assay kit (Thermo Fisher Scientific). RNA integrity was assessed with the Agilent RNA 6000 Pico chip (Agilent) and the Agilent 2100 Bioanalyzer instrument (Agilent). The mRNA of the Inputs was purified using the kit NEBNext Poly(A) mRNA Magnetic Isolation Module (NEB) following the manufacturer’s instructions. The mRNAs of the IP samples were not purified. Library preparation was performed using the NEBNext Ultra II library prep kit for Illumina (NEB) following the manufacturer’s instructions and 8–10 cycles of library amplification. Finally, an equimolar pool was generated with all the samples and sequenced in two 50-nt single-read lanes of a HiSeq 2500 Sequencer (Illumina). Raw FastQ files were aligned against the *X. laevis* genome (UCSC version 9.2, excluding chrUn chromosomes), with Bowtie2 2.2.2 [52], considering 1 mismatch and reporting best alignment site per read. FastQC v011 was used to perform a quality control overview of FastQ files and aligned BAM files. Principal component analysis (PCA) to assess sample similarity using coverage correlations were performed using htSeqTools 1.20 [53]. Binary tracks for all reads in TDF format were generated with IGVTools 2. Default IP versus Input peak calling for enrichment evaluation was performed using MACS 1.4.2 with default options. For differential enrichment gene-based analysis, gene regions were extracted from the Xenbase *X. laevis* 9.2 annotation, using the GenomicFeatures package [54] from Bioconductor. Raw Bowtie2 aligned reads were used to generate gene-level counts, using the options allowMultiOverlap = TRUE, ignoreDup = FALSE, countMultimappingReads = FALSE, minMQS = 1. Afterwards, DESeq2 was used to compare groups [55]. Differentially enriched genes in the IPs versus background were selected using a Benjamini–Hochberg adjusted *P*-value < 0.05 and FC > 2. Adjustment for artifactual log_2_FC due to low count genes was performed with the lfcShrink function from DESeq2. Differential enrichment between CPEB1 and the CPEB2-4 group was performed with DESeq2. Targets of at least one CPEB were considered preferentially enriched based on a lfcShrink log_2_FC > 2 and Benjamini–Hochberg adjusted *P*-value < 0.05 in CPEB1 versus CPEB2, CPEB3, or CPEB4 or the reciprocal comparison.

Functional enrichment analyses of differentially enriched targets were performed with Enrichr [56]. Only significant categories (*P*-adj < 0.05) within the ontologies and pathways sections were retrieved for visualization.

Analysis of 3′ UTR features was performed using the most abundant 3′ UTR per transcript from Yang et al.’s dataset [57]. The distance (in nucleotides) from the polyadenylation signal hexanucleotide (PAS hex) to the 3′ end and 3′ UTR length in targets of any CPEB versus non-targets were quantified using custom Perl scripts as a quality control of the transcripts. 3′ UTR architectures predictive of CPEB regulation were determined with the script published by Piqué et al. [2] with some modifications: the maximum distance between the PAS hex and the 3′ end was relaxed to 60 nucleotides, and non-canonical PAS hex definitions were incorporated [58].

A scan for de novo motifs in CPEB1–4 targets versus non-targets or CPEB1-preferential targets versus CPEB2-4-preferential—and vice versa—was performed with the findMotifs function from the HOMER software [59] with motif lengths of 8, 10 and 12.

### RIP reverse transcription (RT)-qPCR

RNAs were co-immunoprecipitated and eluted from the beads as previously specified. RNAs were purified with simplyRNA Cells Kit (Promega). The RNA was then reverse transcribed with random primers and the RevertAid reverse transcriptase (Thermo Fisher Scientific) following the manufacturer’s instructions. The qPCR was performed in a Quantstudio 6 Flex (Applied Biosystems) using PowerUp SYBRGreen Master Mix (Applied Biosystems) with the transcript-specific primers specified on Additional file 2: Table S4. The enrichment of target sequences in each IP was calculated relative to the Input and NI IP negative controls, to determine if the selected mRNAs were targets. Subsequently, the enrichment in CPEB1 relative to CPEB2, CPEB3, or CPEB4 was calculated to determine the preferential enrichment in one group versus the other, using the delta CT method.

### CPEB1-4 to CPEB1 or CNOT2 co-localization experiments

The mCherry red signal was acquired with the DPSS561 excitation laser set to 12% and the HyD2 detector set to 578-565 nm with a gain of 10%. DAPI and GFP signals were acquired using the aforementioned settings. To assess the extent of co-localization between the red and green channels, the Coloc2 plugin of ImageJ was applied. For each image, Spearman’s correlation coefficient and the Costes *P*-value were obtained.

### Ultrastructure expansion (U-ExM) microscopy and super-resolution imaging

For the experiments, U-ExM cells were seeded on uncoated coverslips. Transfections were performed as previously described.

Ultrastructure expansion of fixed U-2 OS cells was performed following indications of the original protocol [60]. mCherry was stained with goat-anti-mCherry polyclonal (Antibodies-online) 1:50 for 2 h 30’ followed by anti-goat Alexa Fluor 647 (Thermo Fisher Scientific) 1:250 for 2 h 30’. GFP was stained with anti-GFP polyclonal Alexa Fluor 488 (Thermo Fisher Scientific) 1:100 for 2 h 30’ followed by anti-GFP polyclonal Alexa Fluor 488 again at 1:200 for 2 h 30’. Before mounting, the gels were also stained with DAPI 1:2000 for 20’. For image acquisition, a piece of the expanded gel was mounted in a 6-well glass bottom plate (1.5 coverslip, 20 mm diameter from MatTek) coated with poly-l-lysine (Sigma). Super-resolution microscopy was performed using an Elyra PS.1- LSM880 confocal microscope (Zeiss) with an Airyscan mounted. The microscope was equipped with a 100 × 1.46 NA Alpha-Plan Apochromat oil, and the fast-Airyscan module was used for detection. Z-stacks with a 0.247-μm step size were acquired with a XY pixel size of 34 nm. Images were processed using Zen black.

### Live cell imaging of CPEB droplets

Cells expressing GFP-CPEB1–4 were monitored with a Spinning Disk Microscope (Andor Revolution xD, Andor). A total of 800 images were taken per experiment, with a size of 140 × 140 pixels and acquiring two Z-stacks with a slice of 0.5 μm. The typical frame rate was set to 8 images per second (125 ms/frame), and the exposure time of the EMCCD camera (Andor) was set to 50 ms. For acquisition, AOTF 488 nm laser intensity was set to 12%. The fluorescence intensity of fusion events was quantified using the Fiji software. Imaris software was used (https://imaris.oxinst.com) for particle tracking analysis. Images were rescaled setting the voxel size to *x* = 1, *y* = 1, and *z* = 1 units, and particles were segmented using an estimated size of 2 units, automatic quality threshold and local contrasting the diameter obtained from the region border. For track selection, the following parameters were set: maximum frame distance of 6 frames, maximum gap size of 0 frames, and track duration minimum of 7 s. From each track, data regarding particle size, track length, track displacement, and straightness was collected for subsequent analysis.

### 6-Hexanediol treatment

Cells treated with 4% 1,6-hexanediol were monitored using a spinning disk microscope (Andor Revolution xD, Andor). Images were taken every 15 s, with a size of 512 × 512 pixels and acquire 20 Z-stacks with a slice of 0.5 μm. In every experiment, four and fifty images were acquired before and after treatment, respectively, with a 30-s break in between to apply the treatment. For acquisition, AOTF 488 nm laser intensity was set to 7%, and the EMCCD camera was set to an exposure time of 50 ms.

### AlphaScreen protein-nucleic acid interactions assay

*X. laevis* His-tagged CPEB1 and CPEB4 were purified using Ni–NTA Agarose beads (Qiagen) and dialyzed in the storage buffer (25 mM Tris–HCl pH 8, 100 mM NaCl, 5% glycerol, 5 mM MgCl_2_, 10 mM imidazole). Two 3′ biotinylated single-stranded RNA oligonucleotides were synthesized by Integrated DNA Technologies (IDT). The sequences correspond to 28 nucleotides of the cyclin B1 3′ UTR (Accession J03166) and contain a *wt* CPE (CPE-A: AGUGUACAGUGUUUUUAAUAGUUUGUUG) or a CPE with an A to G base substitution at position 17 (CPE-G: AGUGUACAGUGUUUUUGUAAGUUUGUUG).

Amplified Luminescent Proximity Homogenous Assay (Alpha) was performed with AlphaScreen Histidine (Nickel Chelate) detection kit (PerkinElmer) to study the interaction between the RNAs and proteins described above. Optimal concentrations were determined using cross-titration of individual components. To compare the binding affinity, CPE-A and CPE-G RNAs were tested against CPEB1 and CPEB4*.* AlphaScreen assay was performed using two concentrations of protein (50 and 75 nM) and a series of concentrations of RNA (ranging from 0.5 to 75 nM) in a final 40 μL volume of assay buffer (10 mM HEPES pH 7.4, 100 mM KCl, 3 mM MgCl_2_, 1 mM DTT, 0,2 mg/mL tRNA, 0,1% Tween). RNA (10 μL) was incubated with the protein (10 μL) at RT for 1 h. Subsequently, streptavidin donor beads (50 ng/μL final concentration) were added and placed in the dark for 30 min. Then, Ni-Chelate acceptor beads (50 ng/μL final concentration) were added and the final 40 μL of reaction were incubated at RT in the dark for 2 h to reach equilibrium. Plates were sealed with TopSeal–A PLUS adhesive film (PerkinElmer). The kinetics were performed in duplicate. Non-specific binding was determined in the absence of RNA. Assays were performed in triplicate in ½ area white 96-well OptiPlates (PerkinElmer).

Fluorescence signal was detected on an EnSight plate reader (PerkinElmer), and specific interactions were analyzed from nonlinear regression fits of the data according to One-Site Total binding model in GraphPad Prism version 9.1.1.

## Supplementary Information

Additional file 1: Figs. S1–S8. Supporting figures.

Additional file 2: Tables S1–S6. Supporting tables.

Additional file 3. Uncropped figures.

Additional file 4: Review history.

## Data Availability

The RIP-Seq datasets generated and analyzed during the current study are available in NCBI GEO, under the accession GSE189550 [61]. The mass spectrometry proteomics data have been deposited to the ProteomeXchange Consortium via the PRIDE partner repository with the dataset identifiers PXD030419 [62], PXD033349 [63], and PXD030480 [64]. The codes for the RIP-Seq [65] and BioID [66] analyses are available on GitHub. The open source code used for the RIPseq analysis and BioID and stepwise guide for their usage are freely available from the Zenodo website, with the following DOI references: 10.5281/zenodo.6906871 [67] and 10.5281/zenodo.6906859 [68]
