# Supplementary material for: Comparative analyses of vertebrate CPEB proteins define two subfamilies with coordinated yet distinct functions in post-transcriptional gene regulation
Source: Genome Biol. 2022 Sep 12;23:192. doi: 10.1186/s13059-022-02759-y (PMC9465852; doi:10.1186/s13059-022-02759-y)
Supplement: Supplementary file 1 — Additional file 1: Figs. S1–S8. Supporting figures. [file 13059_2022_2759_MOESM1_ESM.pdf]

Figure S1

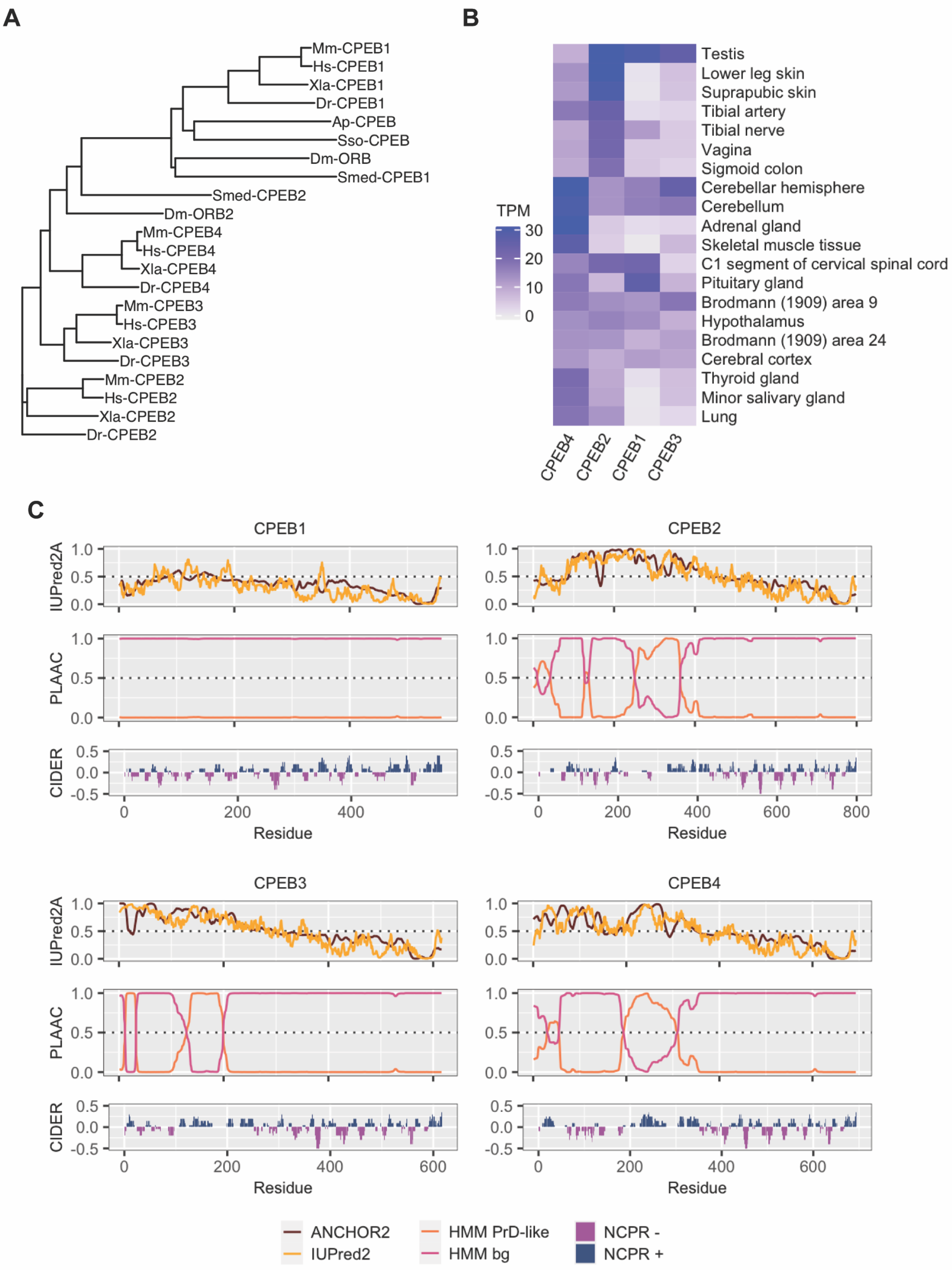

Figure S1. Features of the CPEB-family of RNA-binding proteins. A. Phylogenetic tree (produced with MUSCLE by neighbor-joining without distance corrections) of Orb/CPEB1-4 proteins across species. Mm, Mus musculus; Hs, Homo sapiens; Xla, Xenopus laevis; Dr, Danio rerio; Ap, Aplysia californica; Sso, Spisula solidissima; Dm, Drosophila melanogaster; Smed, Schmidtea mediterranea. B. CPEB1-4 mRNA levels (median TPM, transcripts per million) across human tissues heatmap. The data used for the analyses was obtained from the GTEx Portal on 02/12/2020. For visualization purposes, only the top five tissues for each CPEB have been selected. C. Selected protein sequence features of X. laevis CPEB1-4: IUPred2 and ANCHOR2 scores for disordered protein regions and disordered binding regions, respectively; log likelihood for sequence under background (bg) or prion-like state (PrD-like) under two-state HMM, calculated with PLAAC with default parameters; net-charge per residue (NCPR) calculated with CIDER.

Figure S2

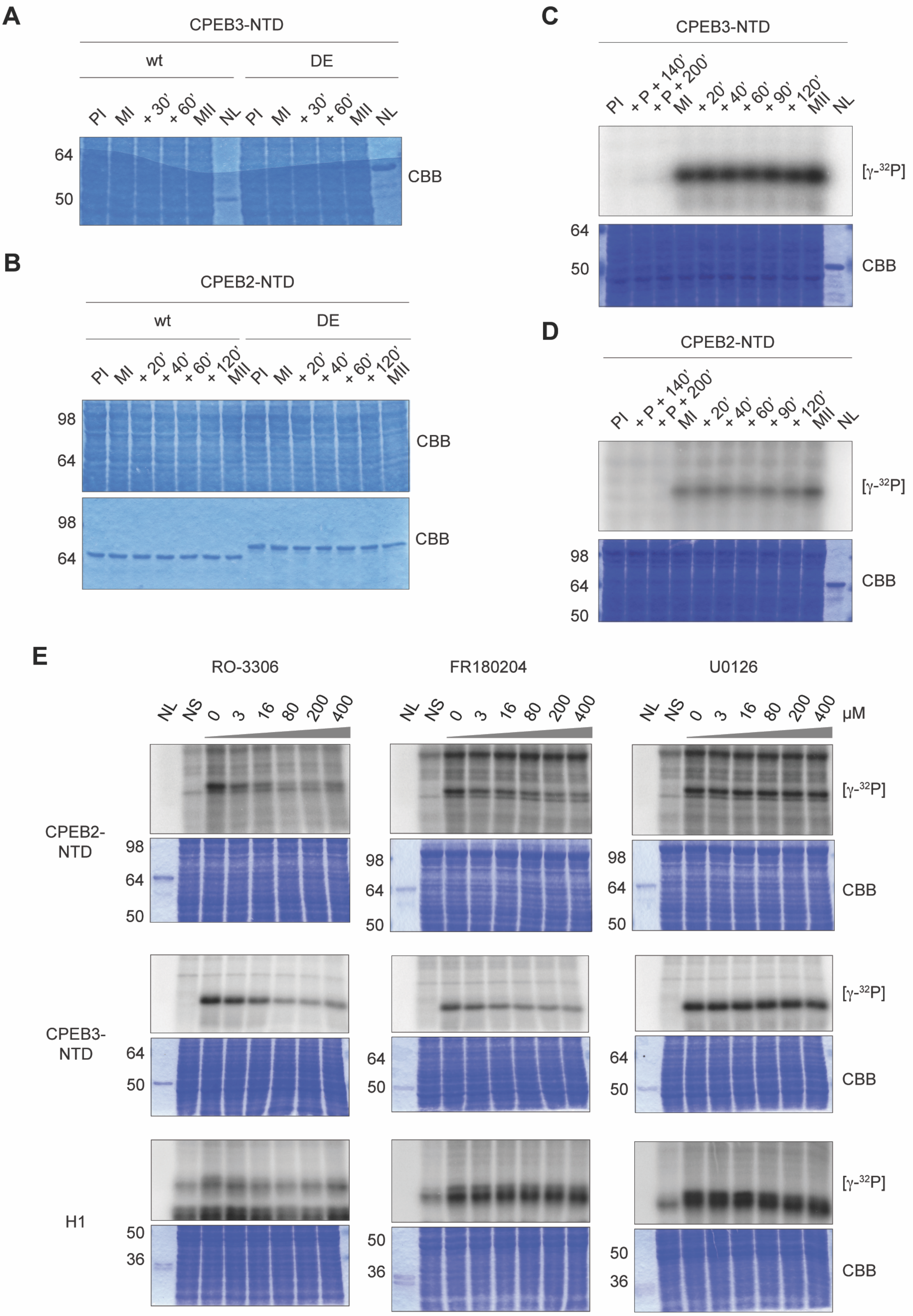

Figure S2. CPEB2 and CPEB3 are phosphorylated by Cdk1 and ERK2 in the meiotic cell cycle. A. Coomassie Brilliant Blue (CBB)-stained gel of meiotic maturation time-courses, related to figure 1F (n = 2). B. CBB-stained gel of meiotic maturation time-courses, related to figure 1I (n = 3). C. [γ-32P]-ATP incorporation by CPEB3-NTD at the indicated maturation time-points (n = 2). D. [γ-32P]-ATP incorporation by CPEB2-NTD at the indicated maturation time-points (n = 3). E. Inhibition of [γ-32P]-ATP incorporation to CPEB2-NTD, CPEB3-NTD or Histone H1 by increasing kinase inhibitor concentrations, related to figure 1L. Representative autoradiographies and CBB, all n between 3 and 4. Abbreviations: wt, wild-type; DE, phosphomimetic mutant; PI, prophase-I; MI, metaphase-I; MII, metaphase-II; NTD, N-terminal domain; NL, no-lysate; NS: no-substrate; CBB, Coomassie-Brilliant-Blue-stained gel; H1, Histone H1.

Figure S3

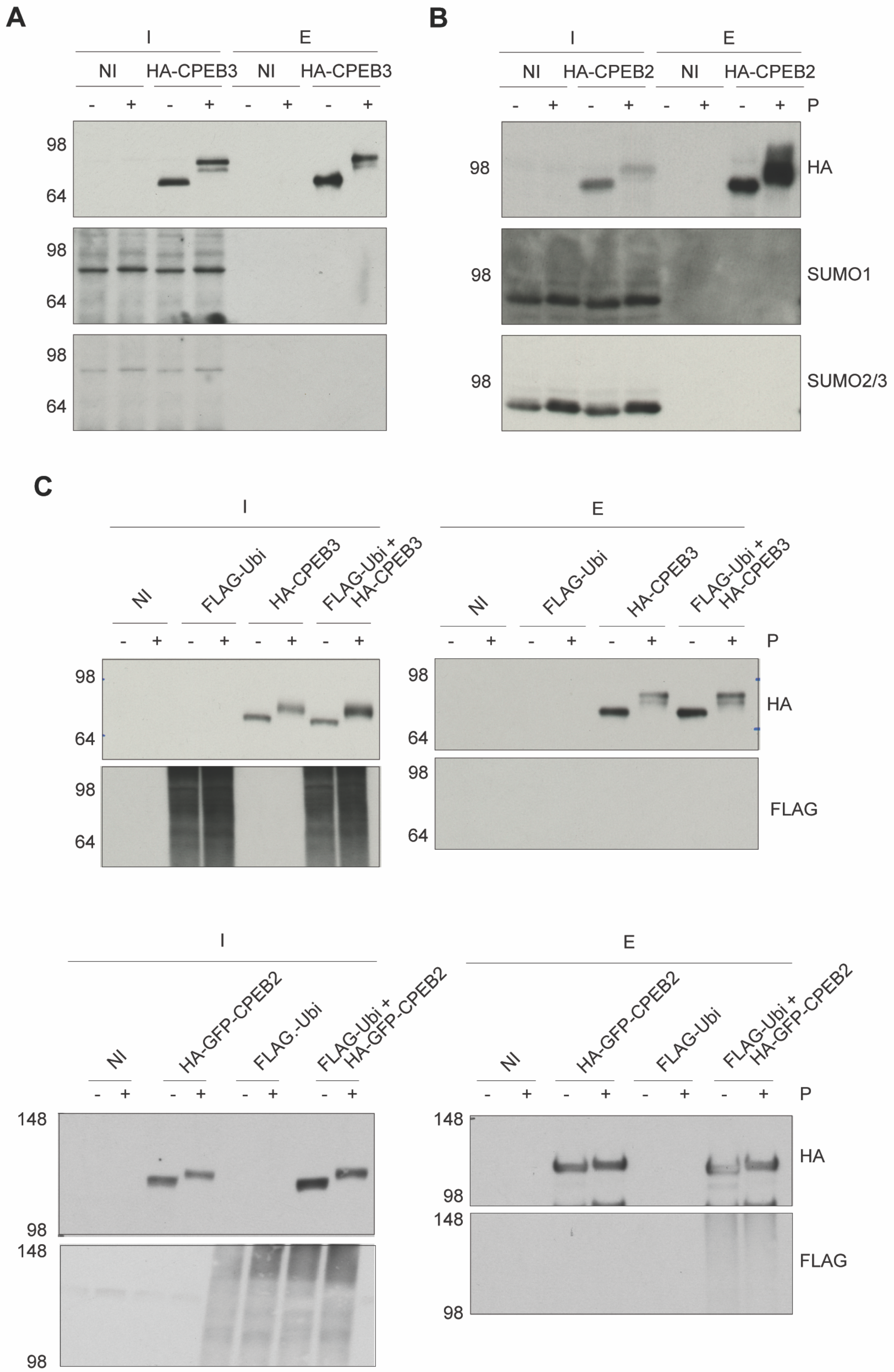

Figure S3. CPEB2 and CPEB3 are not sumoylated or ubiquitylated in the meiotic cell cycle. A. SUMO1 and SUMO2/3 co-immunoprecipitation in HA-CPEB3-overexpressing oocytes (n = 1). B. SUMO1 and SUMO2/3 co-immunoprecipitation in HA-CPEB2-overexpressing oocytes (n = 2). C. FLAG-ubiquitin co-immunoprecipitation (HA IP) in HA-CPEB3 and FLAG-ubiquitin overexpressing oocytes (n = 2). D. FLAG-ubiquitin co-immunoprecipitation (HA IP) in HA-GFP-CPEB2 and FLAG-ubiquitin overexpressing oocytes (n = 2). Note that HA-GFP-CPEB2 was used instead of HA-CPEB2 due to its better detection. Abbreviations: I, input; E, IP eluate; P, progesterone; NI, not-injected.

Figure S4

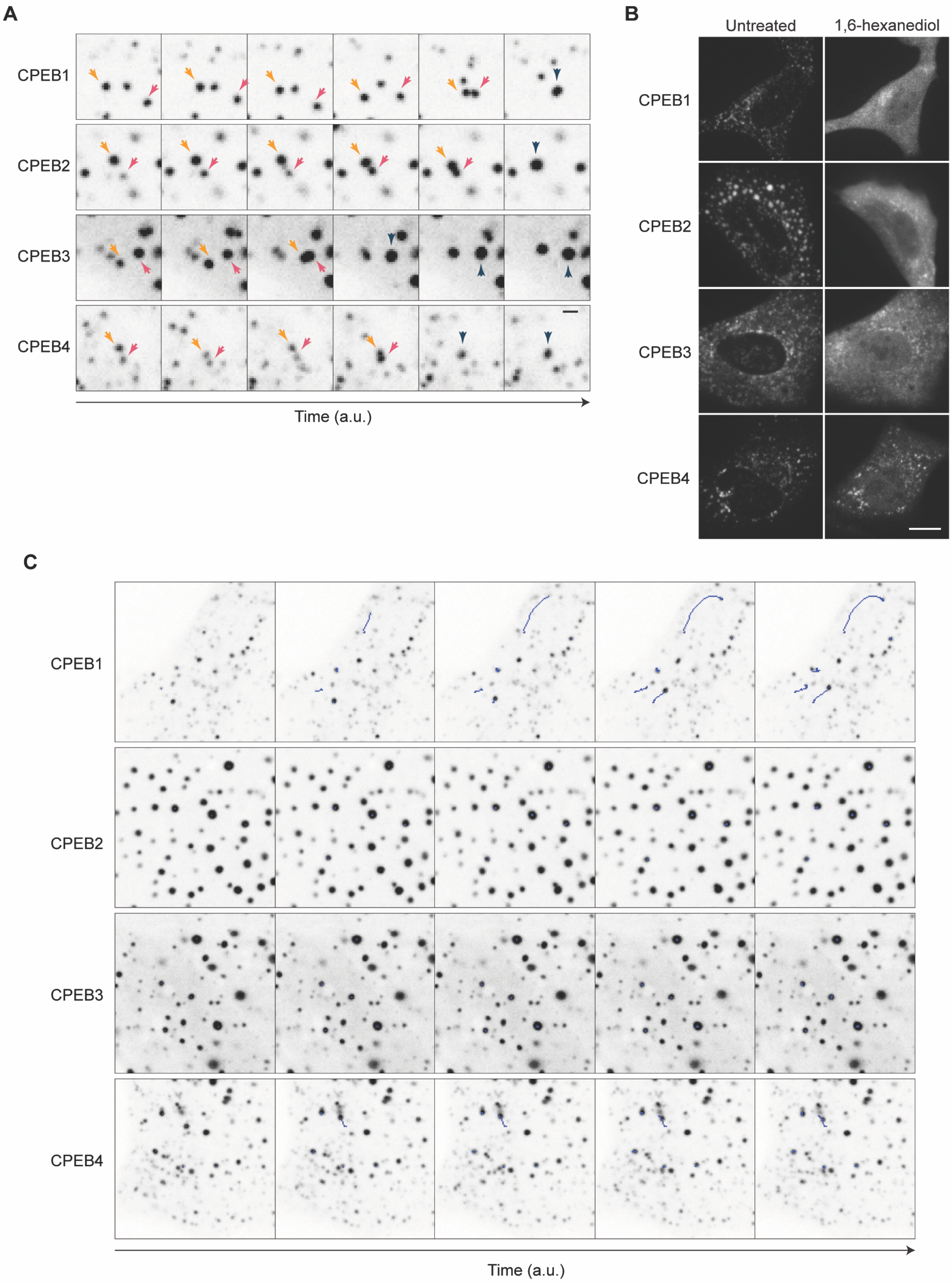

Figure S4. The CPEBs form dynamic cytoplasmic condensates. A. Time course of CPEB1-4-GFP overexpressing live cells whereby fusion events were observed. Scale bar, 1μm. B. CPEB1-4-GFP condensate dissolution upon 4% 1,6-hexanediol treatment (n = 1). Scale bar, 10 μm. C. Time-lapse of the movement of CPEB1-4-GFP. The overlay of the particle tracks is shown in blue.

Figure S5

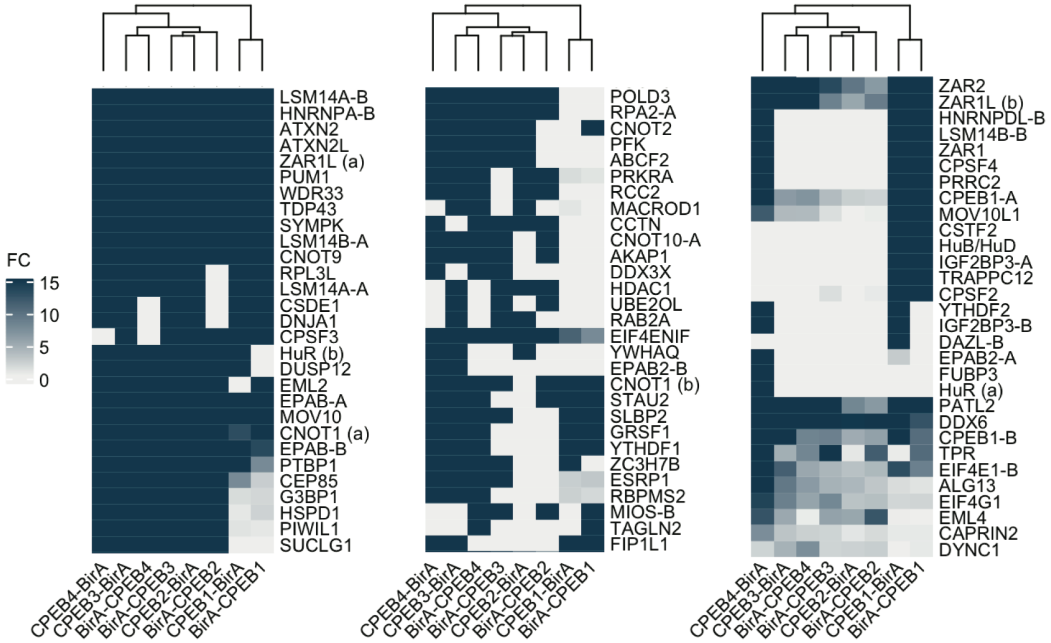

Figure S5. Comparative CPEB1-4 proximome compositions. Clustered heatmap of FC enrichment relative to BirA-alone in the CPEB1-4 proximomes in X. laevis PI-oocytes, determined by BioID (n = 4). Only high-confidence preys of at least one CPEB are displayed across conditions. The clustering was performed using Euclidean distances and complete linkage.

Figure S6

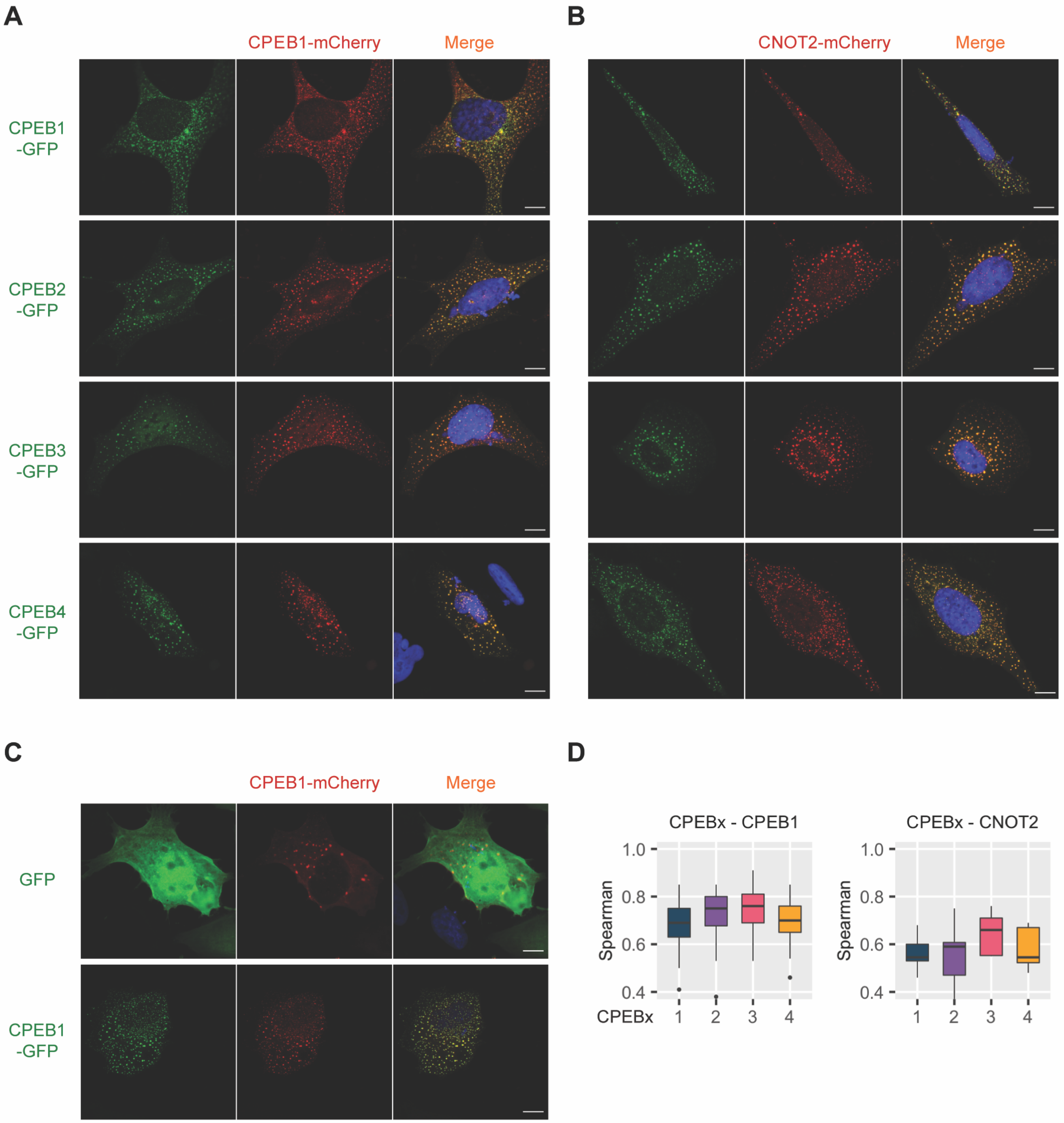

Figure S6. The CPEBs co-localize with CPEB1 and CNOT2. A. Co-localization images of CPEB1-4-GFP with CPEB1-mCherry. CPEB1-GFP, n = 68; CPEB2-GFP, n = 64; CPEB3-GFP, n = 61; CPEB4-GFP, n = 66. B. Co-localization images of CPEB1-4-GFP with CNOT2-mCherry. CPEB1-GFP, n = 10; CPEB2-GFP, n = 10; CPEB3-GFP, n = 10; CPEB4-GFP, n = 10. C. GFP alone with CPEB1-mCherry negative control co-localization. Representative images out of CPEB1-GFP with CPEB1-mCherry, n = 10 and GFP with CPEB1-mCherry, n = 23. The brightness in panels A-C was modified for display, not for quantification, as specified in Table S5. The merge shows the blue channel with DAPI, not shown as a single panel. All scale bars are 10 µm. D. Magnitude of the co-localizations of CPEB1-4-CPEB1 (A) and CPEB1-4-CNOT2 (B), expressed as Spearman's correlation coefficient determined with Coloc2 (see materials and methods).

Figure S7

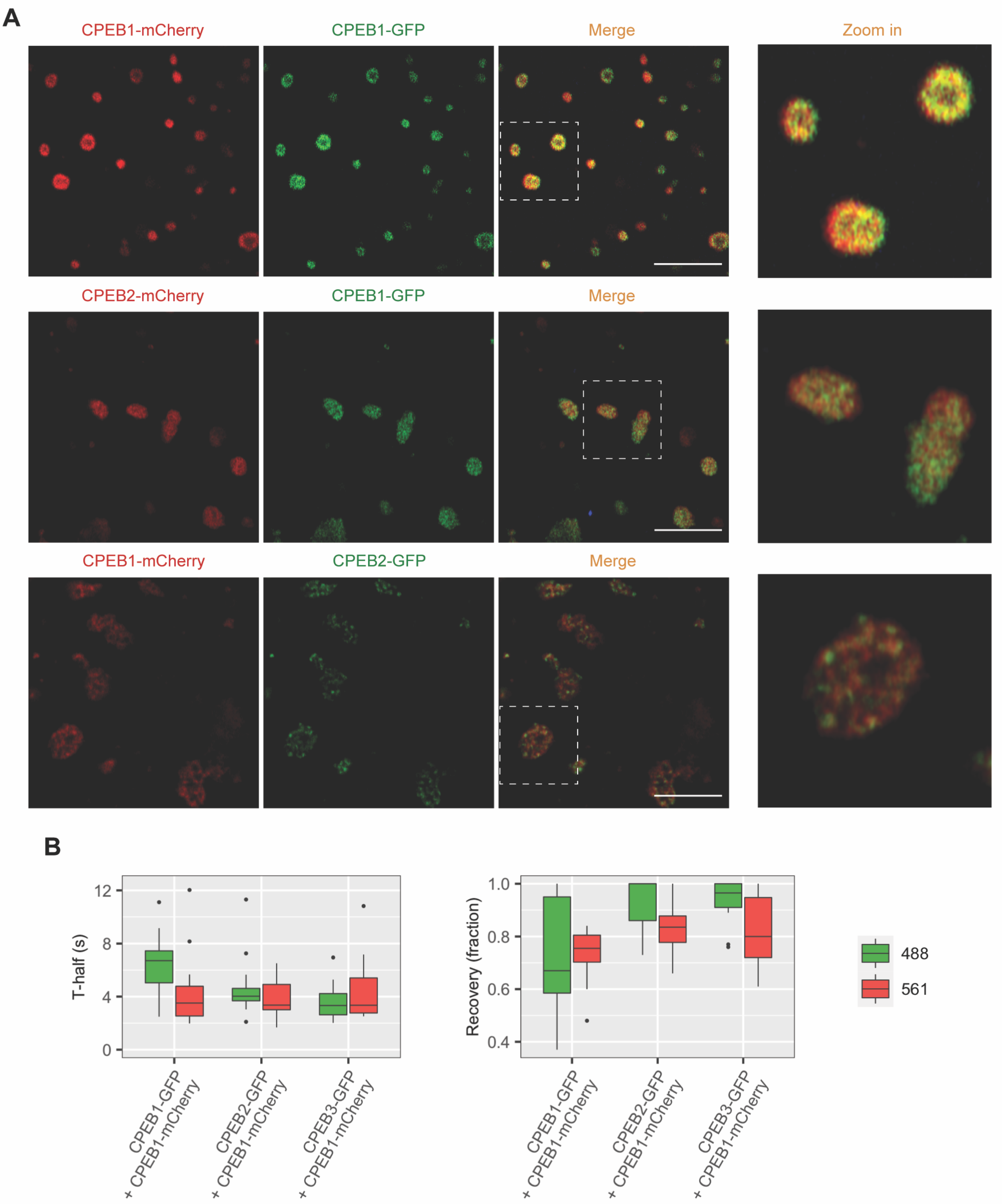

Figure S7. The CPEBs possess distinct properties and occupy a complementary space in droplets. A. Deconvolved confocal image of U-ExM of CPEB condensates in U-2 OS cells, stained with anti-GFP and mCherry antibodies. The brightness was modified for display, equally for all channels and montages, as specified in Table S6. Scale bar, 10 µm. B. Distribution of the half-time of recovery (t-half) and recovery fraction parameters obtained from the FRAP curves of the combinations of CPEB1-3-GFP (green, 488 nm) with CPEB1-mCherry (red, 561 nm). CPEB1-GFP + CPEB1-mCherry, n = 15 and n = 14, respectively; CPEB2-GFP + CPEB1-mCherry, n = 19 and n = 6, respectively; CPEB3-GFP + CPEB1-mCherry, n = 14 and n = 8, respectively.

Figure S8

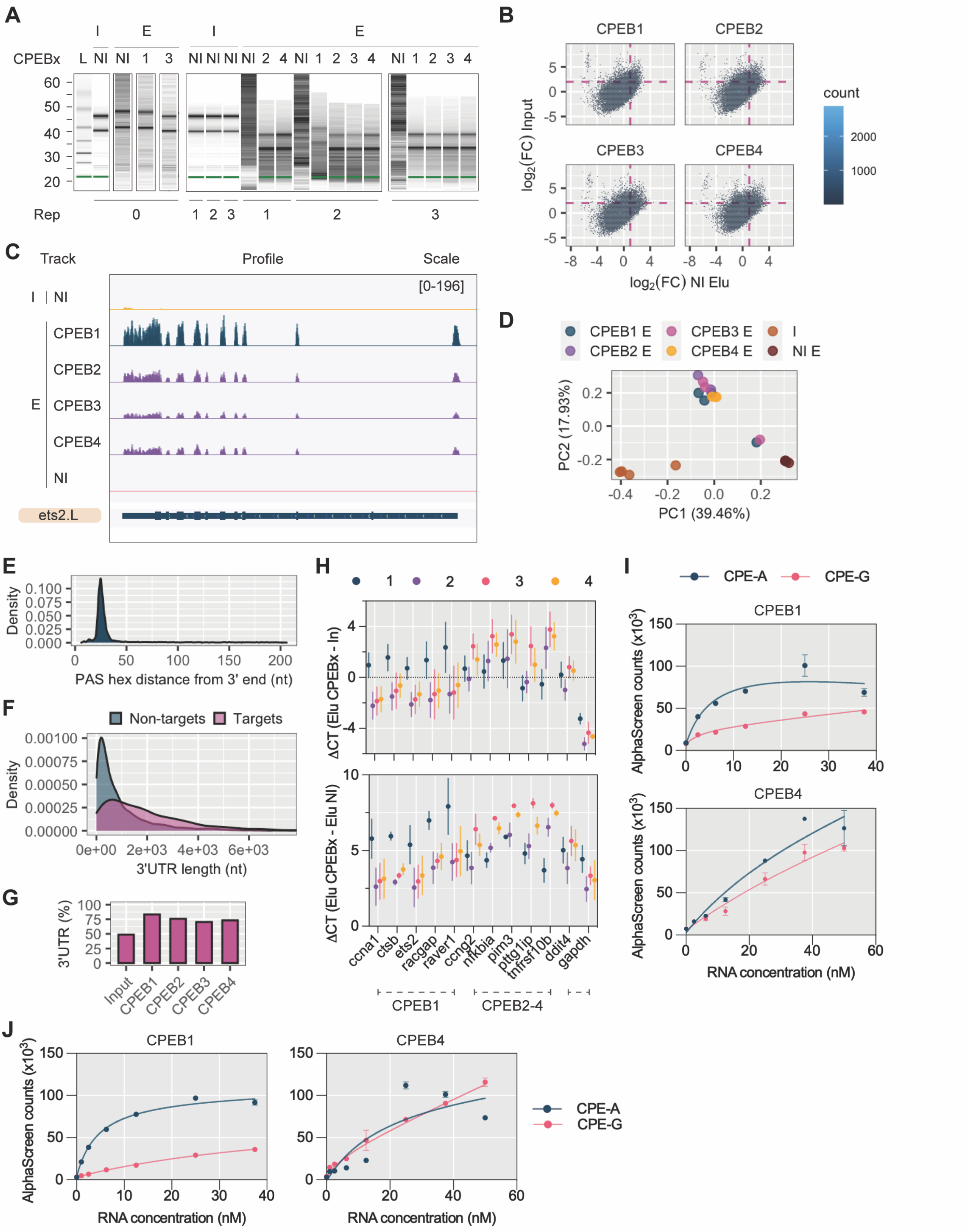

Figure S8. Features of the CPEB1-4 targets defined by RIP-Seq. A. Bioanalyzer electrophoresis of the HA-immunoprecipitated RNA for the CPEB1-4 RIP-Seqs. B. Fold-change enrichment relative to the input versus not-injected elution for all genes in the RIP-Seq experiment. Dashed lines mark the fold-change thresholds that define targets [log2(FC) Input ≥ 2, log2(FC) NI Elu ≥ 1], in addition to

p-adj < 0.05. The number of mRNAs that meet the target definition are 944, 1072, 1089 and 1062 for CPEB1-4, respectively. C. Read mapping of a high-confidence target, ets2.L, across conditions. Each track contains the overlay of all replicates. The read scale – specified on the top right - is the same for all samples. D. Principal component plot of the RIP-Seq samples, showing the first two principal components (PC1, PC2). PC1 and PC2's contribution to total variance is indicated in parenthesis. E. Density plot of the distance, in nucleotides (nt), between the polyadenylation signal hexanucleotide (PAS hex) and the 3' transcript end in the 3'UTR dataset used. F. Density plot of the 3'UTR length in CPEB-targets versus non-targets. G. Percentage of motif architectures predictive of regulation by CPEB1 in the different RIP-Seq target subsets. H. RIP-qPCR enrichment (expressed as delta CT) of indicated candidates relative to the input (top) and to the NI elution (bottom). The candidates are either CPEB1-preferential targets, CPEB2-4-preferential targets or non-targets, separated with dashed lines. Data points represent the mean and standard deviation (n = 3). I and J. AlphaScreen assays of CPEB1 and CPEB4 (50 nM) binding to RNAs containing the CPE-A or CPE-G motifs. Error bars indicate the standard deviation of the technical replicates (n = 2). The experiment was performed in triplicate, one shown in Figure 5F. Abbreviations: L, ladder; NI, not-injected; E or Elu, eluate; I or In, input; P, progesterone; Rep, replicate.
