# Supplementary material for: Comparative analyses of vertebrate CPEB proteins define two subfamilies with coordinated yet distinct functions in post-transcriptional gene regulation
Source: Genome Biol. 2022 Sep 12;23:192. doi: 10.1186/s13059-022-02759-y (PMC9465852; doi:10.1186/s13059-022-02759-y)
Supplement: Supplementary file 3 — Additional file 3. Uncropped figures. [file 13059_2022_2759_MOESM3_ESM.pdf]

Uncropped Figure 1

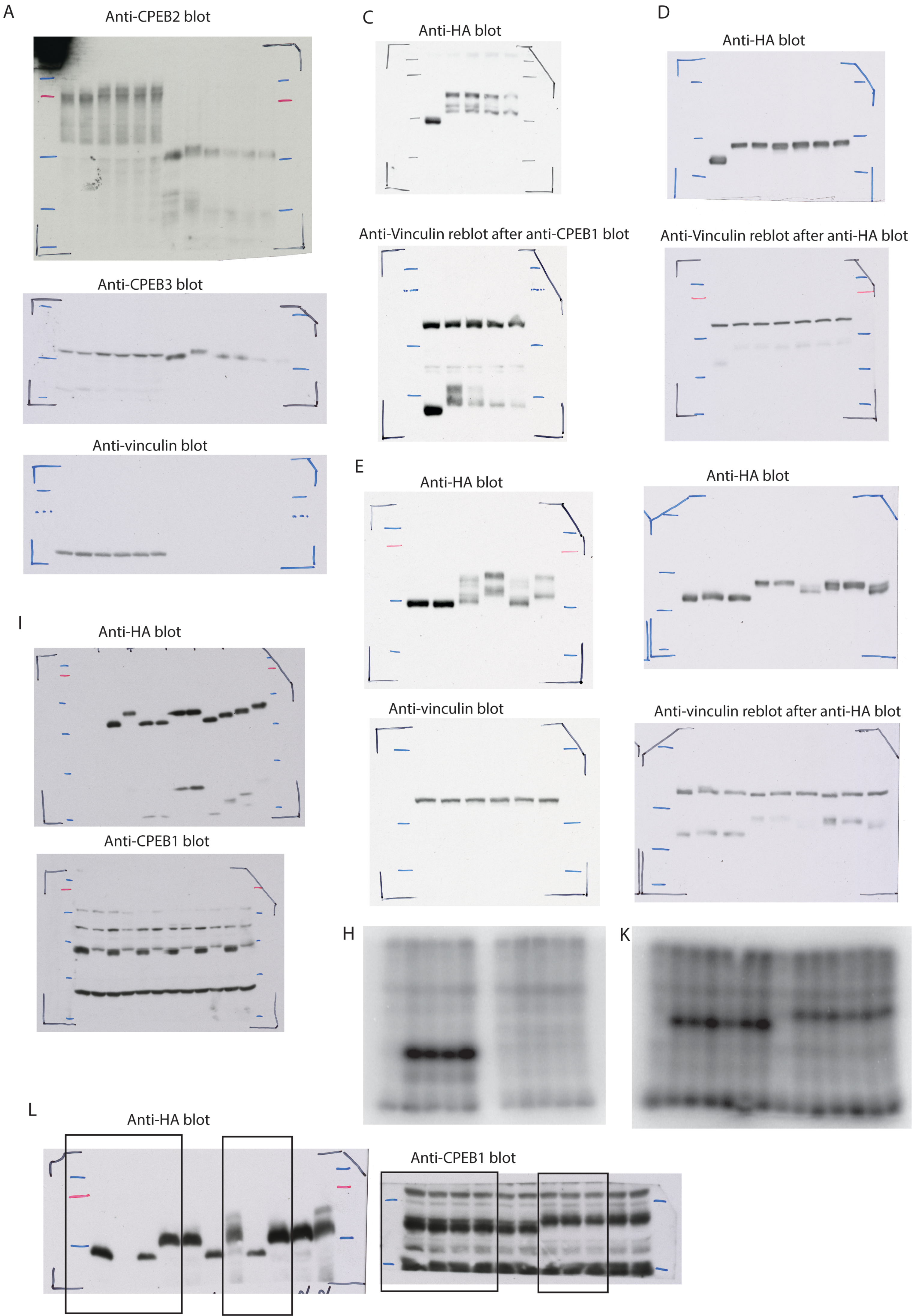

Uncropped Figure 1E

Anti- HA blot

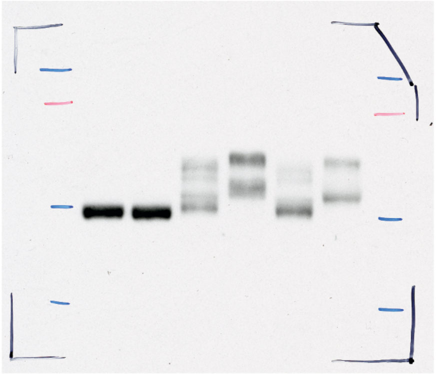

Anti- HA blot

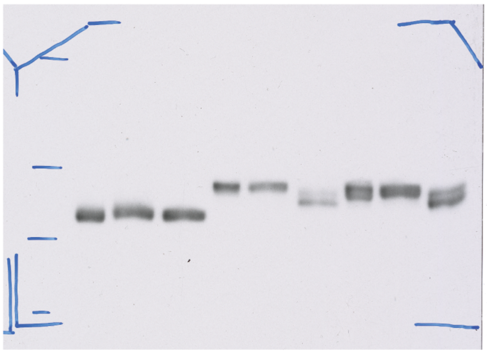

Anti- vinculin blot

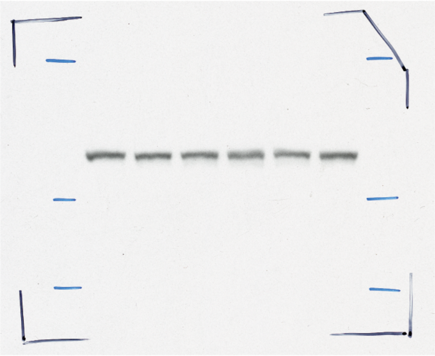

Anti- vinculin reblot after anti-HA blot

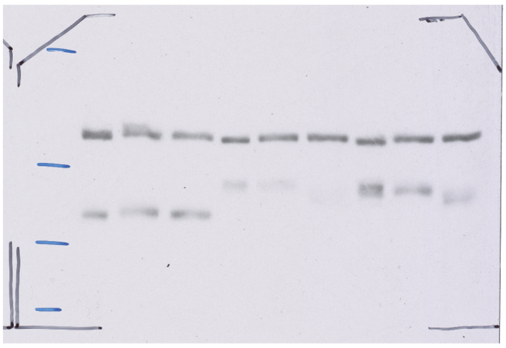

Uncropped Figure 3

A

Anti-CPEB1 blot

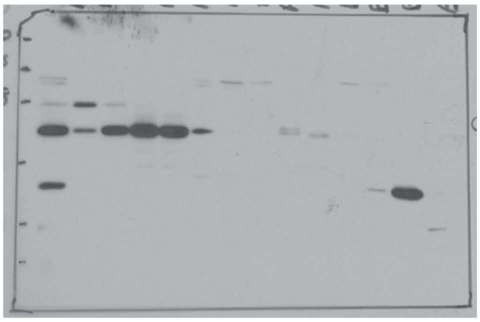

Anti-CPSF2 blot

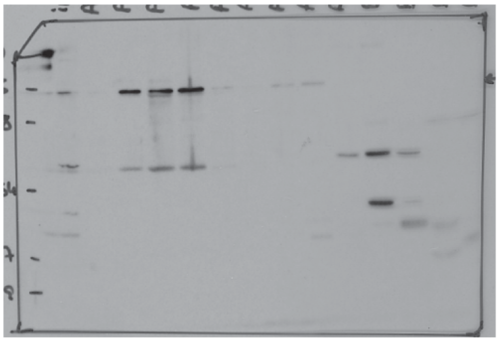

Anti-DDX6 blot

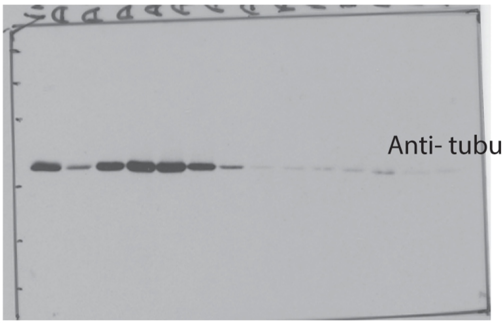

Anti- tubulin blot

Anti-EIF4ENIF blot

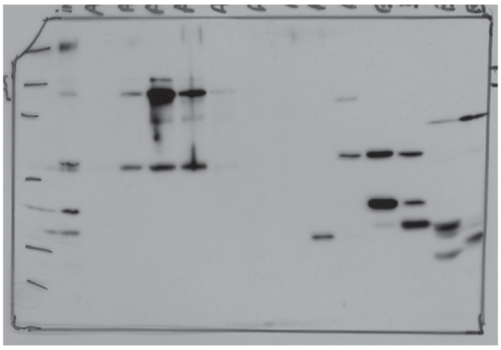

Anti-EIF4E1b blot

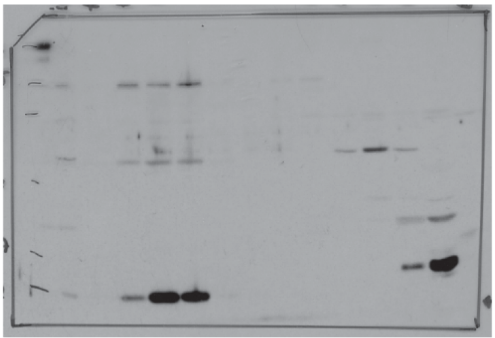

Anti-tubulin blot

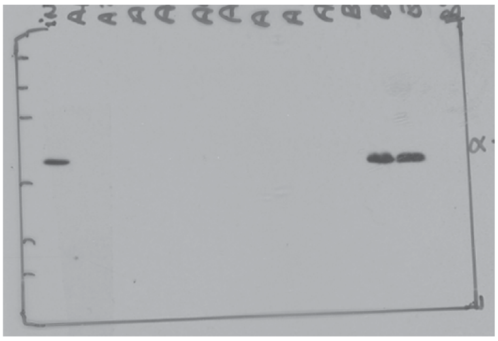

Anti-Maskin blot

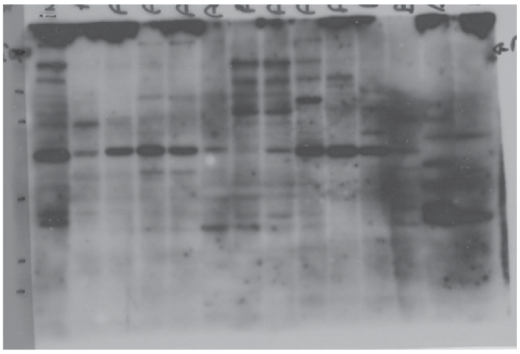

Anti-PARN blot

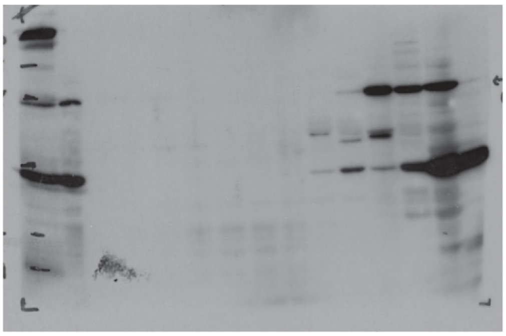

Anti-GLD2 blot

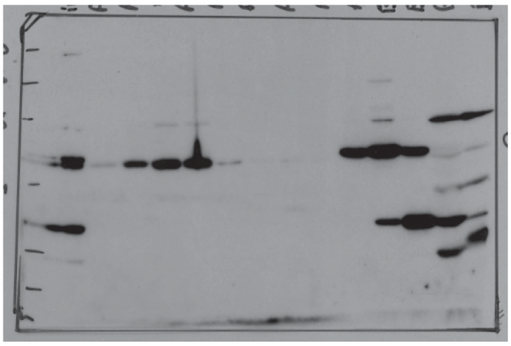

Uncropped Figure 3C

Anti-CPEB1 blot

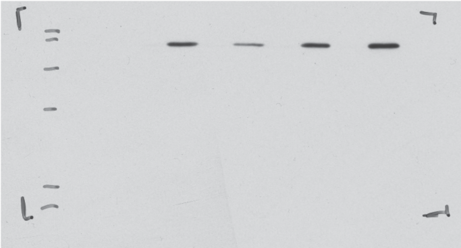

Anti-HA blot

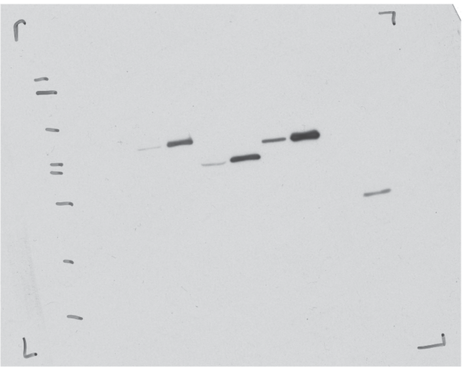

Anti-DDX6 blot

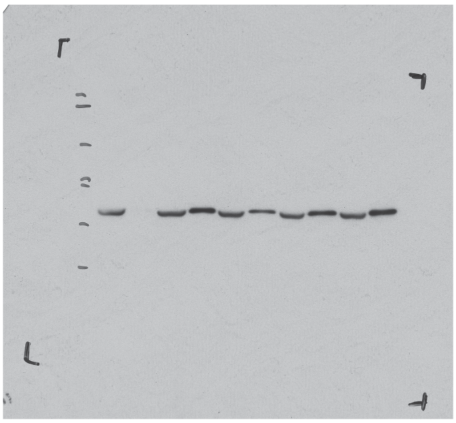

Anti-CPEB1 blot

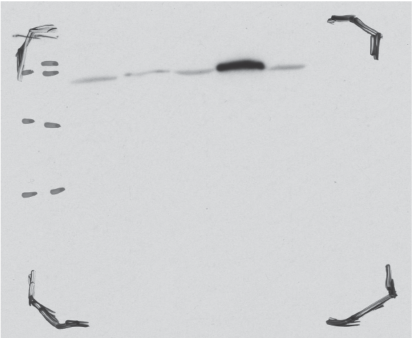

Anti-HA blot

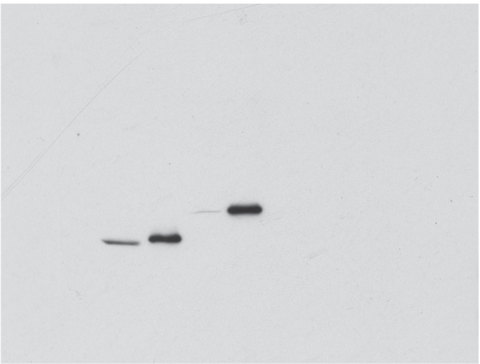

Anti-CPEB1 blot

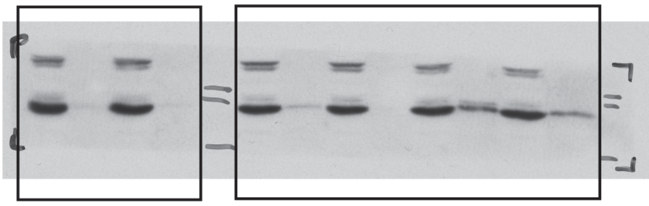

Anti-HA blot

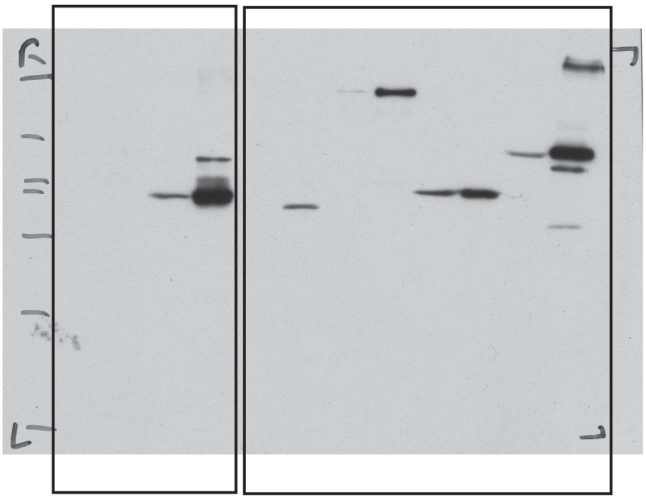

Uncropped Figure 3D

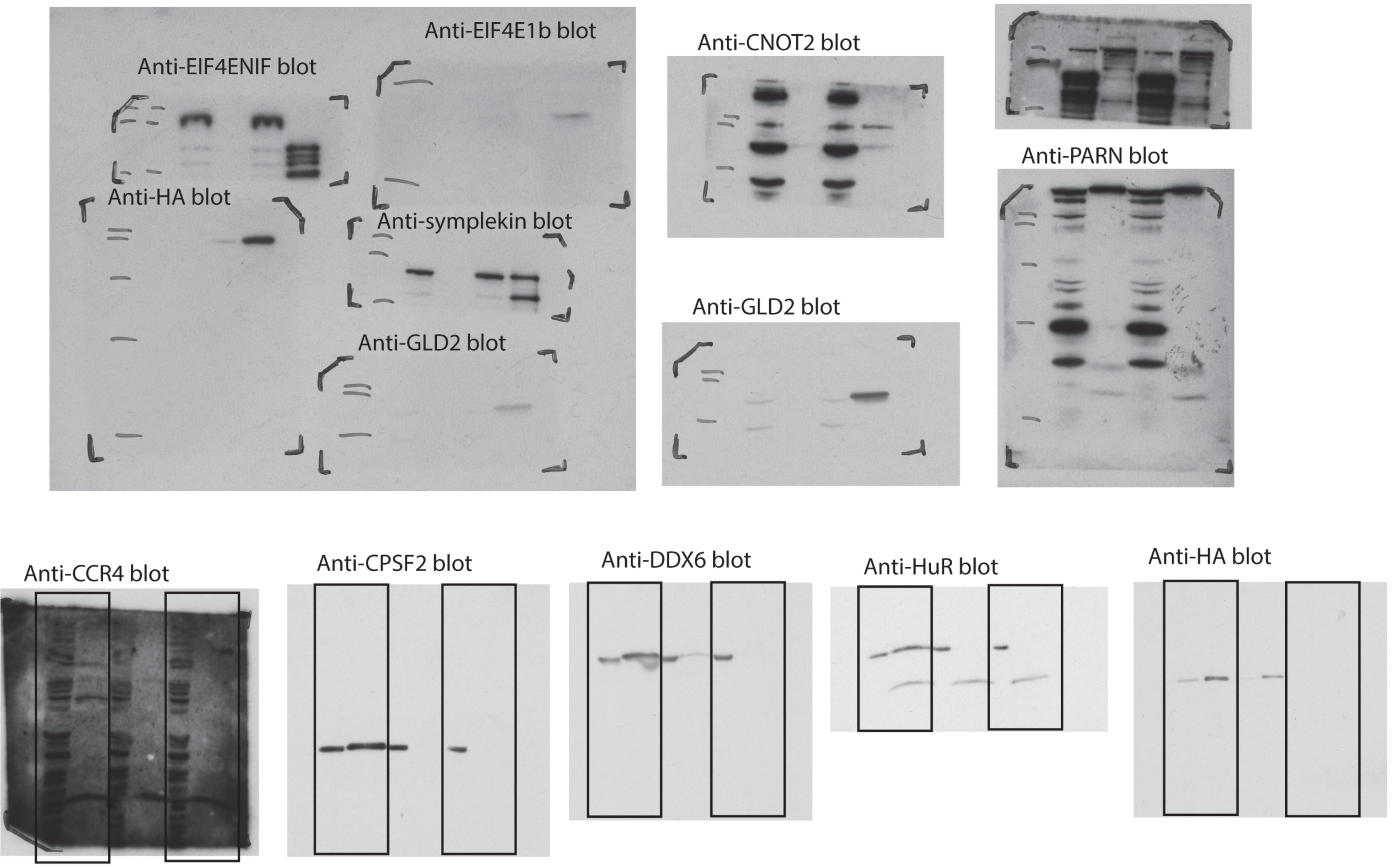

Uncropped Figure 3F

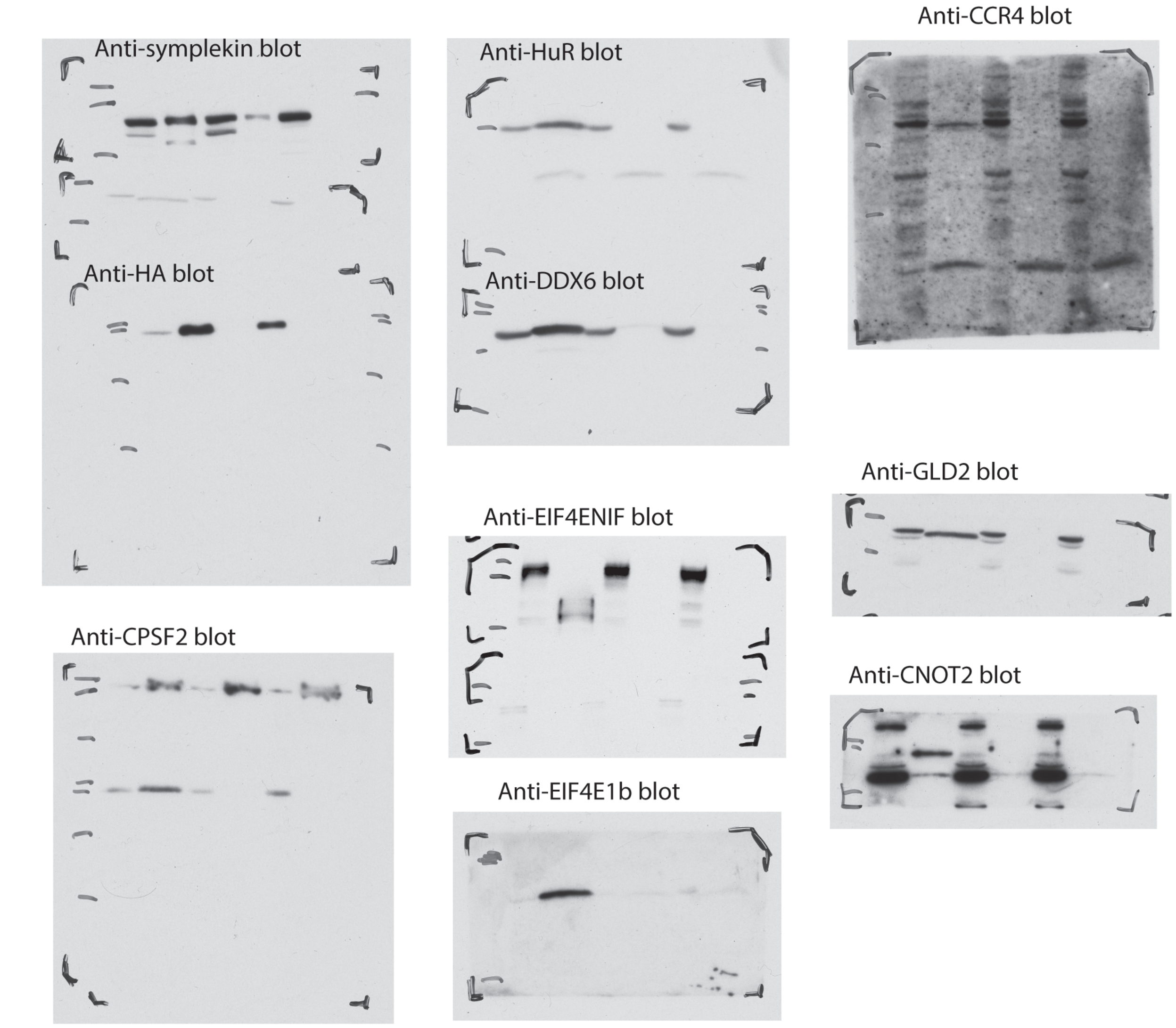

Uncropped Figure 4

D

Anti-symplekin blot

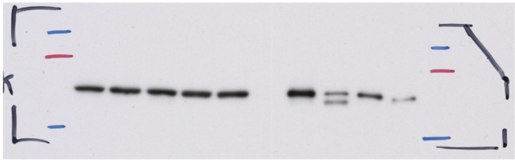

Anti-EIF4ENIF1 blot

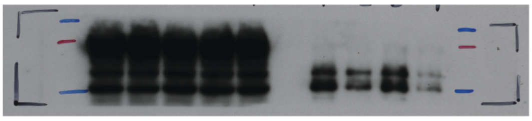

Anti-HA blot

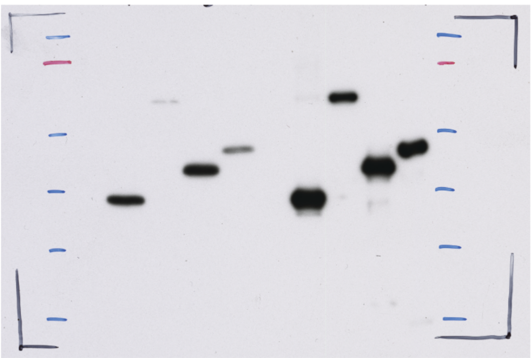

Anti-CPEB1 blot

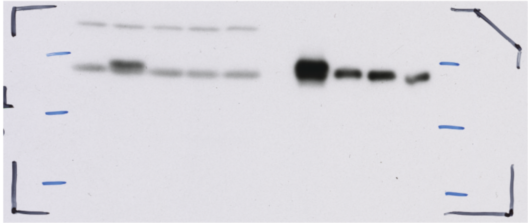

Anti-GLD2 blot

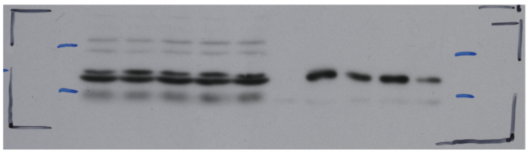

Anti-HuR blot

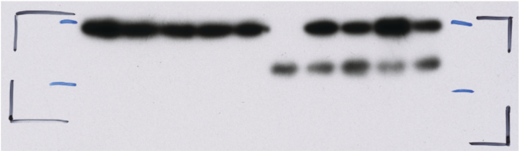

Anti-DDX6 blot

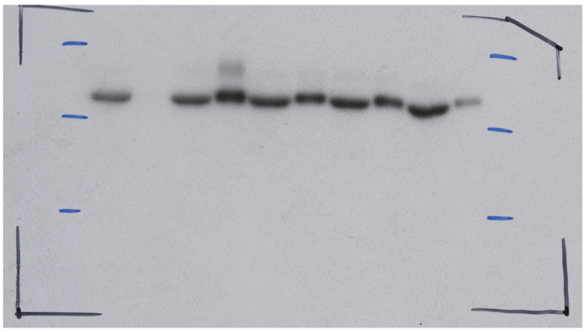

Anti-HA blot

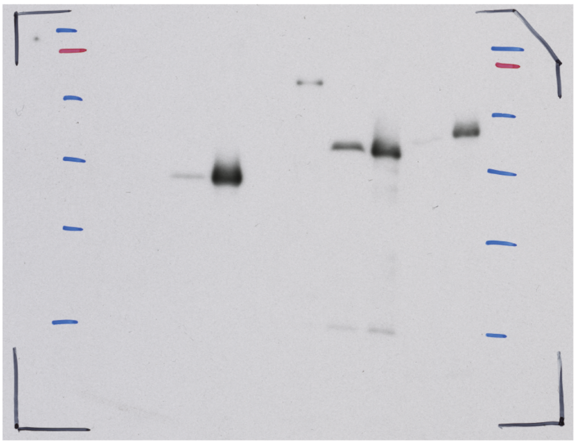

Anti-CPSF2 blot

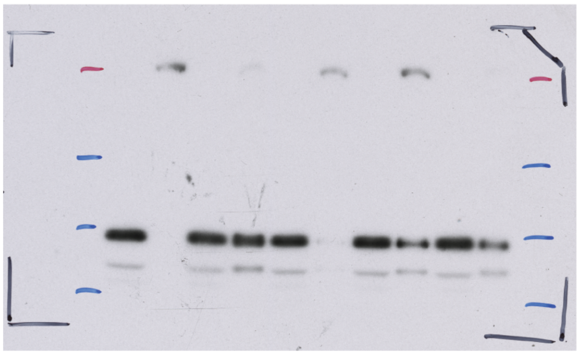

Anti-EIF4E1b blot

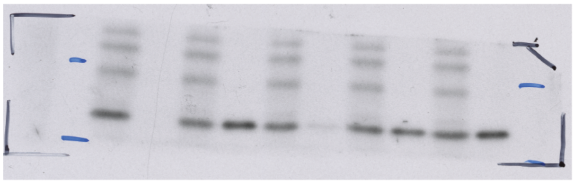

Anti-HA blot

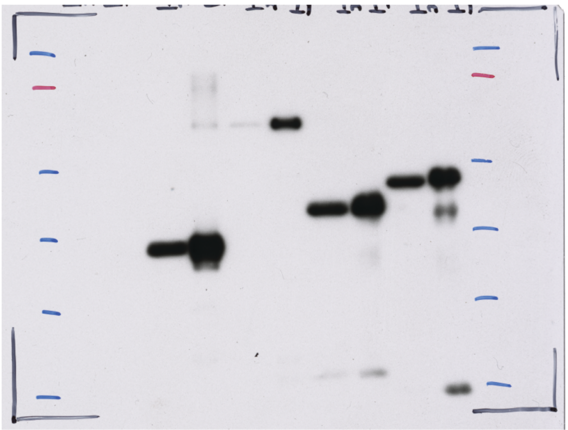

Uncropped Figure 5

A

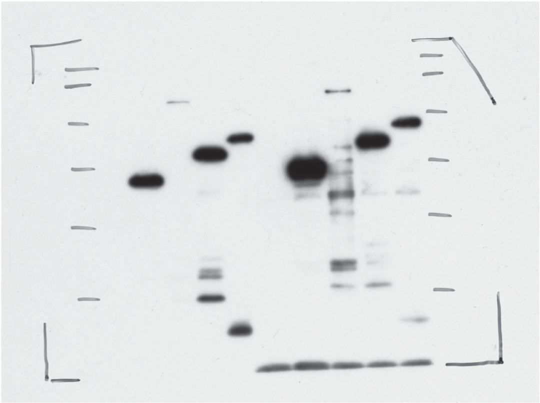

anti-HA blot

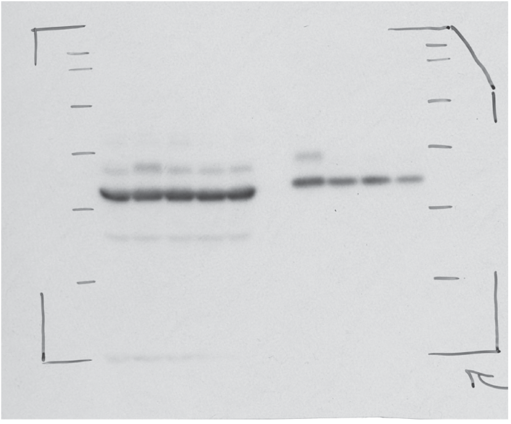

anti-DDX6 blot
