# Supplementary material for: Comparative analyses of vertebrate CPEB proteins define two subfamilies with coordinated yet distinct functions in post-transcriptional gene regulation
Source: Genome Biol. 2022 Sep 12;23:192. doi: 10.1186/s13059-022-02759-y (PMC9465852; doi:10.1186/s13059-022-02759-y)
Supplement: Supplementary file 4 — Additional file 4: Review history. [file 13059_2022_2759_MOESM4_ESM.docx]

Review History

**First round of review**

**Reviewer 1**

**Are you able to assess all statistics in the manuscript, including the appropriateness of statistical tests used?** Yes, and I have assessed the statistics in my report.

**Comments to author:**

In this manuscript Arque et al. in detail characterize the CPEB proteins, which play central roles in regulation of gene expression at the post-transcriptional level. Following studies of their regulation, ability to form assemblies, their interaction partners and their target mRNAs, the authors argue for that CPEB1 and CPEB2-4 represent two distinct entities whose regulation differs and whose downstream effects are both overlapping and distinct. To my knowledge this is the most comprehensive parallel evaluation of the CPEB-proteins which should be of interest to those working on post-transcriptional regulation of gene expression. These studies are both broad (by comparing the 4 CPEBs) but also detailed by e.g. comparing repressive to activated states of CPEB1. Yet, the manuscript is essentially descriptive. In general, the studies are well performed and the data appears to be of high quality. Yet, I recommend performing a limited number of revisions and clarifications as indicated below.

Major:
1. Figure 1B shows the MS based detection of CPEBs. Here it appears that there is only one peptide spectrum which uniquely matches CPEB2. Why is this? The statement that "All four CPEBs were detected by MS/MS" on lines 112 would also benefit from a discussion regarding the seemingly poor detection of CPEB2.
2. It would be interesting to hear whether the authors think that the interactions detected in proximome studies are in one "unit" or whether distinct units.
3. Figure 3F requires a positive control for binding to the Y365A mutant of CPEB1. This would likely be selected from identified binders in figure 3E.
4. In figure 4B it seems that SYMPK does not bind to CPEB2. Yet in figure 4D it seems that it does. Furthermore, CPSF2 is suggested not to bind CPEB2-4 in figure 4B but appears to bind in 4D. This raises concerns regarding the analysis underlying figure 4B which appears to be done using heuristic thresholds while a continuous representation of the data may be more relevant. Nevertheless, it seems that the data for distinct binding between CPEB proteins presented in 4B may be inaccurate and that additional validation of the distinct binding partners identified in figure 4 is necessary. This seems especially important for those commented on in the text such as m6A readers and the set of proteins identified for CPEB2-4 that were not identified for CPEB1.
5. PRRC2, TARDBP, DDX3X etc are suggested to be CPEB3/4 specific partners but they do not seem to be indicated in figure 4B. Why is that? Similar to the point above, should this not be validated?
6. I am a bit confused about the finding that CPEB1 seems to be associating with all CPEBs. What is the implication from this for the other proteins identified in proximome studies in terms of interactions? On line 208 these are referred to as interactors - how is this defined in these studies?
7. When determining shared and specific mRNAs binding to CPEBs it is unclear how these were found. Figure 5B shows a Venn diagram arising from comparing each CPEB to control (?) but overlaps or distinctions in such Venn diagrams are not sufficient to support that transcripts are truly shared or distinct (e.g. one transcript may just pass a threshold in one condition and just miss in a different condition and would then be seen as "unique" in a Venn diagram although if compared using statistical approaches one could not conclude this). Therefore, statistical analyses are required e.g. comparing all CPEBs to control to identify shared binding and e.g. CPEB1 vs CPEB2-4 to identify distinct partners of CPEB1 or CPEB2-4. In the methods it seems that this may have been done but it is not clear. Did you try to identify those that are distinct to each of the CPEBs?

Minor
1. In figure 1G, J. What are the white and grey areas representing?
2. There are issues when referencing to figures in the text. E.g. line 148 "Figure SE".
3. Lines 195, CPSF should be CPSF2
4. When identifying the RNA elements, what background model was used?
5. S7E is better if total area is the same for the different subsets.
6. Line 613: "PCA-like plots". What is this?
7. Line 609 "." Is missing.
8. Line 630 "PAS hex distance" could be better explained.

**Reviewer 2**

**Are you able to assess all statistics in the manuscript, including the appropriateness of statistical tests used?** No, I do not feel adequately qualified to assess the statistics.

**Comments to author:**

Arqué and Cañete et al. Genome Biology.
Comparative analyses of vertebrate CPEB proteins define two subfamilies with
coordinated yet distinct functions in post-transcriptional gene regulation.

Arqué et al. performed a comparative study of the vertebrate CPEB protein family. The study classified the CPEBs into two subfamilies, CPEB1 and CPEB2-4. The authors addressed the importance of CPEBs-NTD in regulating LLDS by phosphorylation. First, they showed how the absence of NTD in CPEB1 and CPEB3 affects the formation and distribution of foci, but not in CPEB2. Furthermore, they showed that CPEB1 undergoes LLDS in the presence of RNA, in an mRNP assembly-fashion, while CEPB2,3,4 undergoes LLDS independent of RNA binding.
Next, the authors explored the interactome of CPEB1 by BioID and validated some hits with immunoprecipitation. They reported some already known interactors but also identified new interactors. Following their previous finding on the RNA binding importance in the CPEB1 aggregate formation, they were able to show that abolishing the RNA binding using a reduced RNA binding capacity-mutant results in a loss of some identified CPEB1-interactors.
After investigating the CPEB1 interactome, the authors performed a comparative BioID to compare the CPEB1 interactome with the other CPEB members. The authors found that all CPEBs share some factors, especially CCR-NOT complex subunits, and have member-specific co-factors. Also, CPEBs tend to interact with each other and to co-localize in the same sub-structures.
Finally, the authors performed a RIP-seq to identify the CPEBs targets and found that, similar to protein interactors, the CPEBs share some common mRNA targets and have their member-specific targets. However, the CPEBs2-4 targets a shared cluster of mRNAs different from the CPEB1 target cluster. The common feature between CPEB1-4 targeted mRNAs is that they have longer 3'-UTR, with discrepancies in motif enrichment between the CPEB1 and CPEB2-4 targets.
The datasets presented are novel and informative; however, the conclusions made by the authors are broadly overstated and should either be softened or substantiated.

Major comments
1. The authors conclude that CPEB recruits the CCR4-NOT complex. How do they reconcile this with their conclusion that CPEB1 co-immunoprecipitation and proximity with CCR4-NOT components are RNA-dependent? Moreover, how do they exclude the reverse possibility that the CCR4-NOT complex recruits CPEB?

2. The authors' conclusion that CPEB recruitment of the CCR4-NOT complex represses translation requires substantial further support. The authors must demonstrate that CCR4-NOT in proximity with CPEB represses translation and show that such repression is CPEB dependent.

3. The authors are aware of Youn et al. 2018 Molecular Cell, which employed Bio-ID of CPEB4 and identified CPEB3, CNOT1, DDX6, and EIF4ENIF1 in its proximity. The authors should emphasize the novelty of their results during the discussion of Youn et al.

4. The authors should demonstrate that CPEB1 and CPEB2-4 have different RNA targets. I suggest doing IP and qPCR to validate the specific targets of CPEB family members.

5. The authors should validate the identified motifs for CPEB1 and CPEB2-4 using an approach such as an RNase protection assay?

6. The authors should support the conclusion that CPEB1 interaction with DDX6, CCR4, CNOT2, eIF4ENIF, eIF4E-1b, CPSF2, and HuR is RNA dependent by demonstrating its loss upon RNAse treatment.

7. The authors do not cite evidence that the known RNA binding domains are the only RNA binding domains of CPEB family members. Moreover, given that other associated proteins, including endogenous CPEB proteins, are known to bind RNA, they cannot conclude that the observed liquid-like droplets do not require RNA. Finally, what evidence supports the conclusion that CPEB is involved in forming liquid-like droplets rather than associating with preformed droplets? Once the authors cite or demonstrate that their truncation mutants lack RNA binding, they could conclude that CPEB binding to RNA is not required for CPEB integration into all liquid-like droplets.

The authors observe the cellular localization of phosphomimetic CPEB family members; they should detail the mutants used. For example, CPEB1 with one mutated residue compared to CPEB2 with 20 mutated residues. The authors should present evidence that all the potential phosphorylation sites of an individual CPEB family member are phosphorylated. They should also show that the diffuse localization of CPEB2-4 upon mutation to include 10s of other negatively charged amino acids is specific to the predicted phosphorylation sites.

9. The analysis of the BioID and RIP-Seq datasets should be made publicly available.


Minor comments
1. Their introduction should introduce Maskin, PARN, and GLD2 to the reader.
2. Do the authors conclude that "ortholog-specific phenotypes when individually depleted" demonstrate CPEB family members have differential functions? Even the individual depletion of paralogs would not show this due to possible complications associated with redundancy.
3. In light of Zaccara et al. 2020 Cell, do the authors conclude that the proximity of CPEB1 with YTHDF1 implicates CPEB1 in translational repression?
4. The authors refer to HuR as HuR and ELAVL1 in their STRING analysis. This should be changed or explained to prevent confusion.
5. How do the authors reconcile their data on CPEB family members having proximity to distinct proteins (F4B) while present in the same complexes (F4D/SF5A)?
6. What are the authors' interpretations of the results presented in SF6A and SF6B? Their conclusion should be explicit. For example, what were the observed differences?
7. Authors must cite the work that shows progesterone promotes the CPEB1-switch from repression to activation.
8. Why are CPEB1, DDX6, and EIF4ENIF not abundant in lower fractions after size fractionation? Are these proteins only present in massive foci?
9. What proportion of CPEB is in liquid-like droplets? What is the concentration of CPEB in liquid-like droplets?
10. Do DDX6 and EIF4ENIF have roles in translational repression in CPEB activation complexes?
11. The headings throughout the manuscript should state the conclusion of the following section. In addition, greater emphasis should be given to the narrative that CPEB family members form two distinct but overlapping subfamilies. Finally, the justification of each following technique should be made clear; together, these changes will reduce the misinterpretation that the authors have linked together disconnected datasets.
12. In the first section of results "Meiotic maturation as a model for coordinated expression and regulation of all four CPEBs", except for MS/MS, the authors showed the data only for CPEB2-3 and relied on the previously published data for CPEB1,4 to compare with. It would have been more convincing to see CPEB1 and CPEB4 in the same experiment, instead of referring to a 1994 paper to compare.
13. The inputs in CPEB2 immunoprecipitation (Figure 1A and Figure S3-B) are not visible.
14. In Figure 5E, the authors are stating "We did not find any specific functional category for CPEB1, On the other hand, G1-S cell cycle control and TNF-alpha signaling were enriched among CPEB2-4 targets". The p-value for NOTCH1 signaling pathway enrichment is p<0.05 in CPEB1-targets and <0.01 in CPEB2-4 targets. Why there is a focus on G1-S and TNF-alpha pathways, even though they are less enriched (yet significant)?
15. There are a couple of mislabeling in supplementary figures:
* Figure S3-D IP-E figure: inversion of FLAG-Ubi with HA-GFP-CPEB2
* Figure S6-A, if the FRAP is performed on the same confocal slides, then the labeling in S6-A panel 2 should be CPEB1-mCherry and CPEB2-GFP instead of CPEB2-mCherry and CPEB1-GFP, and in panel 3, should be CPEB3-GFP instead of CPEB2-GFP, otherwise, it doesn't make sense.

**Authors Response**

**Point-by-point responses to the reviewers’ comments:**

Reviewer #1
In this manuscript Arque et al. in detail characterize the CPEB proteins, which play central roles in regulation of gene expression at the post-transcriptional level. Following studies of their regulation, ability to form assemblies, their interaction partners and their target mRNAs, the authors argue for that CPEB1 and CPEB2-4 represent two distinct entities whose regulation differs and whose downstream effects are both overlapping and distinct. To my knowledge this is the most comprehensive parallel evaluation of the CPEB-proteins which should be of interest to those working on post-transcriptional regulation of gene expression. These studies are both broad (by comparing the 4 CPEBs) but also detailed by e.g. comparing repressive to activated states of CPEB1. Yet, the manuscript is essentially descriptive. In general, the studies are well performed and the data appears to be of high quality. Yet, I recommend performing a limited number of revisions and clarifications as indicated below.

***We thank the reviewer for his/her comments and specific points. We have included the requested clarifications as specified in the point-by-point responses.***

Major
1. Figure 1B shows the MS based detection of CPEBs. Here it appears that there is only one peptide spectrum which uniquely matches CPEB2. Why is this? The statement that "All four CPEBs were detected by MS/MS" on lines 112 would also benefit from a discussion regarding the seemingly poor detection of CPEB2.

***The number and quantity of unique peptides for each CPEB is affected by the high sequence identity within the CPEB2-4 subfamily – which is high compared with CPEB1 - and therefore the number of potential unique peptides is lower. In addition, the expression of CPEB1 in oocytes is much higher than for the CPEB2-4 subfamily (in agreement with proteomics studies by Peuchen et al. 2017, and Wuhr et al. 2014). Within the CPEB2-4 subfamily, it appears that CPEB2 is the least abundant of the three, in agreement with its lower stability. It would be safe to assume that CPEB2-4 are proportionately represented in the common peptides, following the ratios of their individually unique peptides.***

***We have included a brief explanation in the revised manuscript.***

2. It would be interesting to hear whether the authors think that the interactions detected in proximome studies are in one "unit" or whether distinct units.

***We agree with the reviewer that “complex-heterogeneity” is a relevant and valid point in any study addressing complexes’ composition, and not easy to get a definitive answer just in one study. For RNA binding complexes the mRNA molecule brings an additional layer of complexity as the proteins in an mRNP potentially include several protein-complexes independently interacting with an mRNA.***

***As shown in Figure 3A, 99% of CPEB1 comigrates in the same molecular weight (12-14 MDa, representing a single mRNP or different mRNPs of similar size). The size of this large mRNP is not sensitive to RNase treatment, indicating that, at least once formed, it is stable through protein-protein interactions. The integration of the CPEB1 interactome with other interactomes (Figure 4C) is very consistent with two interconnected “nodes”. One represented by CPSF and CSTF and the other by the cap-binding complex (eIF4F), PABP and CCR4-NOT, possibly interconnected through Symplekin, a direct CPEB1-interactor. Thus, at least for these two nodes the most plausible explanation is that they are all part of the same mRNP, consistent with the size from Figure 3A.***

***Therefore, we would argue that the most likely scenario is that the cores of the CPEBs proximomes correspond to a single mRNP. We have briefly mentioned this argument in the revised manuscript.***

3. Figure 3F requires a positive control for binding to the Y365A mutant of CPEB1. This would likely be selected from identified binders in figure 3E.

***We apologize for not having indicated this in the original submission. The positive control is Symplekin (SYMPK) that is a direct interactor of CPEB1 (Barnard et al. 2004) and part of the proximome. In the first submission’s immunoblot, SYMPK band was a bit faint, we have therefore changed it by an independent replicate where the SYMPK band is more visible. Thus, SYMPK interacts with WT and Y365A CPEB1 in both BioID and co-IP. We have briefly mentioned this control in the revised manuscript.***

***As shown on ‘Point by Point’ Figure 1, A was Figure 3E in the first submission, while B is the panel present in the reviewed version.***

4. In figure 4B it seems that SYMPK does not bind to CPEB2. Yet in figure 4D it seems that it does. Furthermore, CPSF2 is suggested not to bind CPEB2-4 in figure 4B but appears to bind in 4D. This raises concerns regarding the analysis underlying figure 4B which appears to be done using heuristic thresholds while a continuous representation of the data may be more relevant. Nevertheless, it seems that the data for distinct binding between CPEB proteins presented in 4B may be inaccurate and that additional validation of the distinct binding partners identified in figure 4 is necessary. This seems especially important for those commented on in the text such as m6A readers and the set of proteins identified for CPEB2-4 that were not identified for CPEB1.

***We thank the reviewer for highlighting these apparent contradictions, which arise from aspects that we have failed to explain with enough clarity. On the one hand, we would like to highlight the differences from an “interaction” detected by BioID or by co-IP, two approaches that complement each other but are not identical in their interpretations.***

***As shown in the figure representing a possible CPEB-complex (see ‘Point by Point’ Figure 2), at an approximate scale, modeling the CCR4-NOT complex with the published positions of DDX6 and AGO (Ukleja et al. 2016) and a possible location of CPEB fused to BirA at the N- (red) or C- (orange) termini together with an “endogenous” CPEB (green). BirA biotinylates proteins in a radius of 10-15 nm (inner and outer dashed line circles in the figure), this would include only biotinylation of the closest complex components, whether or not they physically interact. It would not label other complexes independently binding the same mRNA. Weak or transient interactions could be detected by BioID. In contrast, co-IP would presumably immunoprecipitate the whole complex (which is 12-14 MDa; Figure 3A) including long-range components and intermediate proteins. While direct and indirect interactions would be captured, weak and transient interactions would be excluded due to the stringent co-IP conditions. Thus, it is not surprising to find differences between both techniques for large complexes and, rather than failed validation, differential detections probably reflect the architecture of the complex.***

***On the other hand, we thank the reviewer for highlighting some limitations of the way we chose to represent our datasets. In figure 4B we represented the list of hits, i.e. of high-confidence preys, of each CPEB. We defined targets – as explained in the figure legend and materials and methods sections – based on conservative thresholds, commonly used in similar studies (to which we have compared our results), and we used the targets defined in this way for downstream analyses such as functional enrichment, network analysis (Figure 4B) and validations (Figure 3C-D and 3F-H, Figure 4D). For these kinds of comparisons/validations a binary outcome is required. Moreover, the visualization displayed in Figure 4B allowed us to summarize the targets of each CPEB, regardless of the fusion, resulting in a more compact visualization. We agree that in order to facilitate a more quantitative interpretation of the data, a continuous representation would be more suitable. Thus, we have included a heatmap of the fold-change enrichment of each condition relative to the BirA-only negative control in Figure S5. We also encounter some limitations in the continuous representation, such as infinite fold-change values due to missing data in the control condition (common in BioID and, more generally, proteomics datasets).***

***Last, for the double analyses, BioID/co-IP, we focused on the candidates that addressed the controversy of the repression complexes. We agree that other candidates in this list, such as the m6A readers, open interesting possibilities for novel (common and/or specific) functions of the CPEB complexes that deserve more detailed analyses in the future, but we hope the reviewer will agree with us that this goes beyond the scope of this (already quite extensive) study.***

5. PRRC2, TARDBP, DDX3X etc are suggested to be CPEB3/4 specific partners but they do not seem to be indicated in figure 4B. Why is that? Similar to the point above, should this not be validated?

***We apologize for the mistake and for misleading the reader. For each experimental approach we have used the nomenclature of the corresponding database or, for the westerns and published candidates, the one in the referenced manuscripts. That has caused the nomenclature discrepancies for some proteins between Figure 4B and Figure 4C (e.g. TARDBP, which in 4B was referred to as TDP43, or RQCD1, which was otherwise referred to as CNOT9). We have now corrected the names so that they are consistent throughout the paper.***

***In addition, in the network plot, proteins that were not previously known to interact with other proximome components would be disconnected nodes and are not included, since the point is to stress the previously known connectivity among the identified components.***

6. I am a bit confused about the finding that CPEB1 seems to be associating with all CPEBs. What is the implication from this for the other proteins identified in proximome studies in terms of interactions? On line 208 these are referred to as interactors - how is this defined in these studies?

***Assembly of the “repression complex” requires two CPEs at a distance between 0 and 50 nucleotides, which suggest that these complexes are assembled on a CPEB dimer (Pique et al. 2008) The cross-labeling between CPEBs would indicate that it can be a heterodimer. Given that the relative abundance of CPEB1 is much higher than for the other CPEBs, this heterodimer will often include endogenous CPEB1. As depicted in the figure above, the 10-15 nm biotinylating-distance constraint will favor labelling of the Bir1A-CPEB interactors. Interactors in this context refers to biotinylated proteins (proximity-interactors/proximome-components).***

***We have now clarified the term in the main text.***

7. When determining shared and specific mRNAs binding to CPEBs it is unclear how these were found. Figure 5B shows a Venn diagram arising from comparing each CPEB to control (?) but overlaps or distinctions in such Venn diagrams are not sufficient to support that transcripts are truly shared or distinct (e.g. one transcript may just pass a threshold in one condition and just miss in a different condition and would then be seen as "unique" in a Venn diagram although if compared using statistical approaches one could not conclude this). Therefore, statistical analyses are required e.g. comparing all CPEBs to control to identify shared binding and e.g. CPEB1 vs CPEB2-4 to identify distinct partners of CPEB1 or CPEB2-4. In the methods it seems that this may have been done but it is not clear. Did you try to identify those that are distinct to each of the CPEBs?

***We fully agree with the reviewer. That is a general limitation of the Venn diagrams, which, on the other hand, allow for easy visualization of large amounts of information. The Venn diagram was included to illustrate the broad overlap among the different CPEB1-4 targets subsets. The suggested approach was employed to identify targets differentially bound by CPEB1 versus CPEB2-4, which were determined by differential enrichment analysis with DESeq2 (Figure 5C). The corresponding explanation in the materials and methods section and in the main text has been clarified. Both the datasets and the analysis pipeline have been made publicly available.***

***Given the high similarity between the CPEB2-4 subsets, illustrated in Figure 5C and as shown in the clustered heatmaps of Pearson correlation coefficients (see ‘Point by Point’ Figure 3) the distance measure is the value of the Pearson correlation, correlations are shown in the enrichment to the Input – In – and NI IP – NI), we focused on differences between CPEB2-4 as a group, relative to CPEB1; i.e. targets of any CPEB that were differentially enriched in CPEB2, CPEB3 or CPEB4 relative to CPEB1. Within the subset of 1798 targets of any CPEB, 234 mRNAs were tar¬gets of CPEB1 and preferentially CPEB1-regulated, 414 were targets of CPEB2, CPEB3 or CPEB4 and preferentially CPEB2-4-regulated and 1148 were not differentially regulated in either group. We did not try to identify targets unique to CPEB2, CPEB3 or CPEB4.***

Minor
1. In figure 1G, J. What are the white and grey areas representing?
***The white areas represent the proteins’ N-terminal domain, while grey areas represent the C-terminal domain. This information has now been added to the corresponding figure legend.***

2. There are issues when referencing to figures in the text. E.g. line 148 "Figure SE".
***This was a mistake that has now been amended, it should have said “Figure S 2E”.***

3. Lines 195, CPSF should be CPSF2
***CPSF on line 195 has been changed to CPSF2.***

4. When identifying the RNA elements, what background model was used?
***In former Figure 5D (now in Figure 5E), the RNA motifs were identified against the RNA-Seq input sequences. In Figure S7G (now in Figure 5E), the groups of preferential targets were compared against each other, thus the background models are CPEB1- or CPEB2-4-preferential targets, respectively.***

***For clarity, these figures have now been merged on Figure 5E and reference to the background model has been included.***

5. S7E is better if total area is the same for the different subsets.
***The area was the same for both subsets, however, outliers that are not shown in the plot but are considered for the area size calculation made it not seem like that. We have reviewed the data and updated the figure after eliminating a number of outliers, so that now the areas are equivalent.***

6. Line 613: "PCA-like plots". What is this?
***Principal component analysis (PCA) was performed as a quality control to assess the similarity between RIP-Seq samples. The plot had unintentionally been left out, it can now be found on Figure S8D.***

7. Line 609 "." Is missing.
***This was indeed an omission and has now been fixed.***

8. Line 630 "PAS hex distance" could be better explained.
***This refers to the distance from the polyadenylation signal hexanucleotide (PAS-hex) to the 3’UTR end. This was used as a quality measure of the 3’UTR dataset. The plot that the text referred to had been unintentionally omitted but can now be found on Figure S8E.***


Reviewer #2
Arqué and Cañete et al. Genome Biology.Comparative analyses of vertebrate CPEB proteins define two subfamilies withcoordinated yet distinct functions in post-transcriptional gene regulation. Arqué et al. performed a comparative study of the vertebrate CPEB protein family. The study classified the CPEBs into two subfamilies, CPEB1 and CPEB2-4. The authors addressed the importance of CPEBs-NTD in regulating LLDS by phosphorylation. First, they showed how the absence of NTD in CPEB1 and CPEB3 affects the formation and distribution of foci, but not in CPEB2. Furthermore, they showed that CPEB1 undergoes LLDS in the presence of RNA, in an mRNP assembly-fashion, while CEPB2,3,4 undergoes LLDS independent of RNA binding. Next, the authors explored the interactome of CPEB1 by BioID and validated some hits with immunoprecipitation. They reported some already known interactors but also identified new interactors. Following their previous finding on the RNA binding importance in the CPEB1 aggregate formation, they were able to show that abolishing the RNA binding using a reduced RNA binding capacity-mutant results in a loss of some identified CPEB1-interactors. After investigating the CPEB1 interactome, the authors performed a comparative BioID to compare the CPEB1 interactome with the other CPEB members. The authors found that all CPEBs share some factors, especially CCR-NOT complex subunits, and have member-specific co-factors. Also, CPEBs tend to interact with each other and to co-localize in the same sub-structures.
Finally, the authors performed a RIP-seq to identify the CPEBs targets and found that, similar to protein interactors, the CPEBs share some common mRNA targets and have their member-specific targets. However, the CPEBs2-4 targets a shared cluster of mRNAs different from the CPEB1 target cluster. The common feature between CPEB1-4 targeted mRNAs is that they have longer 3'-UTR, with discrepancies in motif enrichment between the CPEB1 and CPEB2-4 targets. The datasets presented are novel and informative; however, the conclusions made by the authors are broadly overstated and should either be softened or substantiated.

***We thank the reviewer for his/her comments and specific points. We have included the requested clarifications/experiments as specified in the point-by-point responses. We have also modulated some the conclusions following the reviewer indications.***

Major
1. The authors conclude that CPEB recruits the CCR4-NOT complex. How do they reconcile this with their conclusion that CPEB1 co-immunoprecipitation and proximity with CCR4-NOT components are RNA-dependent? Moreover, how do they exclude the reverse possibility that the CCR4-NOT complex recruits CPEB?

***While the analysis of the “end-point” assembled complex does not provide information on the order of events, we would argue that CPEBs provide the mRNA target specificity (which probably starts co-transcriptionally as CPEBs are nucleocytoplasmic shuttling proteins) while CCR4-NOT contributes the enzymatic activity. CPE-containing mRNAs are deadenylated in early meiosis (Pique et al. 2008). Consequently, point mutation of this cis-acting element or CPEB-depletion prevent deadenylation. The question was, how can CPEBs, which have no enzymatic activity, promote deadenylation? What we find here is that the mRNP assembled by CPEBs on CPE-containing mRNAs includes, among other proteins, the CCR4-NOT complex. This finding is further supported by the co-submitted manuscript by Poetz et al. where they also find that CPEB4 binds to CCR4-NOT. To the best of our knowledge, the CCR4-NOT complex, lacking sequence specificity, is recruited to its target mRNAs through other proteins that provide the target specificity (Pumilio, miRNA-machinery, etc.). It is also common that assembly of mRNPs is nucleated, or is greatly enhanced, by the mRNA (in many cases co-transcriptionally) even for proteins that interact directly (Perales and Bentley 2009). Altogether, the most logical scenario would be that CPEBs bind the CPE-containing mRNAs and then recruit CCR4-NOT (probably stabilized by non-sequence-specific interactions of CCR4-NOT with the mRNA). For mRNPs that require RNA to promote the protein-protein interactions, in some cases the interactions remain stable even following RNA-digestion whereas in other cases the RNA would be required to stabilize weak protein-protein interactions.***

2. The authors' conclusion that CPEB recruitment of the CCR4-NOT complex represses translation requires substantial further support. The authors must demonstrate that CCR4-NOT in proximity with CPEB represses translation and show that such repression is CPEB dependent.

***That CPEB represses translation through cytoplasmic deadenylation of CPE-containing mRNAs has been shown in numerous studies before (reviewed in Weill et al. 2012), what was unclear was which protein contributed with the deadenylase activity. Indeed, PARN was previously proposed as the deadenylase recruited by CPEB (Kim and Richter 2006). Now we show that PARN is not present in the complex (Figure 3A-D), but instead we detect CCR4-NOT associated with all four CPEBs. Considering that CPEB is required for deadenylation and translational repression and that CCR4-NOT on its own does not have CPE-specificity, we would argue that the published experiments address this question and that there would be little more that we could do beyond repeating these published experiments, many from our own group. Going beyond what is already published would require mapping the interaction domains in the CPEBs and CCR4-NOT and replacing the endogenous proteins (which is almost impossible in the oocyte model) with mutants that fail to interact. We hope the reviewer will agree that that constitutes a project on its own and should be addressed in future studies.***

3. The authors are aware of Youn et al. 2018 Molecular Cell, which employed Bio-ID of CPEB4 and identified CPEB3, CNOT1, DDX6, and EIF4ENIF1 in its proximity. The authors should emphasize the novelty of their results during the discussion of Youn et al.

***We have expanded the previous comment on the findings by Youn et al.***

4. The authors should demonstrate that CPEB1 and CPEB2-4 have different RNA targets. I suggest doing IP and qPCR to validate the specific targets of CPEB family members.

***Following the suggestion by the reviewer we have performed HA-CPEB1-4 RIP-qPCR. We chose 10 mRNAs that are abundantly expressed, 5 of them from the CPEB1-preferentially bound category (ccna1, ets2, raver1, ctsb and racgap1) and another 5 from the CPEB2-4 preferentially bound category (ccng2, nfkbia, tnfrsf10b, pim3, pttg1ip.1). gapdh and ddit4 mRNAs were used as a control of no binding to CPEBs (non-targets). Indeed, the RIP-qPCR confirmed that the selected mRNAs were targets (enriched over the NI IP and Input controls, Figure S8H) and that they were preferentially enriched in the CPEB1 or CPEB2-4 IPs according to the preference determined by RIP-Seq (Figure 5D). Both RIP-Seq and RIP-qPCR validate the differential specificity of CPEBs for the selected targets.***

***These results are now included in Figures 5D and S8H.***

5. The authors should validate the identified motifs for CPEB1 and CPEB2-4 using an approach such as an RNase protection assay?

***Following the suggestion by the reviewer we have validated the motif specificity of CPEB1 and CPEB4 for A- or G-CPEs using AlphaScreen technology. AlphaScreen is a bead-based proximity assay, whereby the tagged protein of interest is bound to acceptor beads and the RNA of interest is bound to donor beads. Only when the donor and acceptor beads come in close proximity, the donor bead excitation results in acceptor bead light emission, therefore, interaction between a given RNA and a protein can be measured by light emission. These results have been added to Figures 5F and S8I. The approach shows that CPEB4 binds equally well to A- or G-CPEs, if anything it binds better to the novel non-canonical G-CPEs than to the canonical A-CPEs. On the contrary, CPEB1 binds better to the canonical A-CPEs than to the non-canonical G-CPEs.***

6. The authors should support the conclusion that CPEB1 interaction with DDX6, CCR4, CNOT2, eIF4ENIF, eIF4E-1b, CPSF2, and HuR is RNA dependent by demonstrating its loss upon RNAse treatment.

***Following the suggestion by the reviewer, we have done the co-IPs with RNase digestion, which are shown in new Figure 3G-H. For some of the interactors - EIF4ENIF1, DDX6, CPSF2, HuR, EIF4E1b - not only the assembly of the complex requires RNA, but also the stability once assembled. For the CCR4-NOT complex, although the assembly of the complex requires RNA, once bound CPEB-NOT interaction is stable enough to resist coimmunoprecipitation even after RNA digestion. As detailed in point 1, both behaviors are common in mRNPs.***

7. The authors do not cite evidence that the known RNA binding domains are the only RNA binding domains of CPEB family members. Moreover, given that other associated proteins, including endogenous CPEB proteins, are known to bind RNA, they cannot conclude that the observed liquid-like droplets do not require RNA. Finally, what evidence supports the conclusion that CPEB is involved in forming liquid-like droplets rather than associating with preformed droplets? Once the authors cite or demonstrate that their truncation mutants lack RNA binding, they could conclude that CPEB binding to RNA is not required for CPEB integration into all liquid-like droplets.

***We thank the reviewer for raising these points in need of further clarification, which we have now expanded. That the binding of CPEBs to- CPE-containing-mRNAs is mediated by the two RRMs and not by the intrinsically disorder N-terminal regions is derived from the experiments in Afroz et al. 2014 and in Guillen-Boixet et al. 2016. This is the common and expected behaviour of all the analyzed RNA-binding proteins containing this typical distribution of IDRs and RRMs. Indeed, to the best of our knowledge, not even in the RNA-interactome capture studies that have identified systematically all canonical and non-canonical RNA-binding proteins, an IDR binding to RNA has never been found.***

***That the IDR of CPEBs can assemble LLDs in the absence of RNA, or of any other protein, is derived from the experiments in Guillen-Boixet et al. 2016. In particular from the fact that the purified N-terminal region of CPEB4 alone can assemble LLDs. This fact has not been detected for CPEB1. Altogether these evidences indicate that CPEBs 2-4, which contain IDRs, can assemble droplets whereas CPEB1, which does not contain such clear IDRs, requires RNA binding for its integration into droplets that contain CPEB2-4. In vivo it is possible that droplets of different origin fuse into heterogeneous superstructures and that is a very active field of research on its own. We have added the appropriate citations in the revised manuscript.***

8. The authors observe the cellular localization of phosphomimetic CPEB family members; they should detail the mutants used. For example, CPEB1 with one mutated residue compared to CPEB2 with 20 mutated residues. The authors should present evidence that all the potential phosphorylation sites of an individual CPEB family member are phosphorylated. They should also show that the diffuse localization of CPEB2-4 upon mutation to include 10s of other negatively charged amino acids is specific to the predicted phosphorylation sites.

***The mutants are indicated in the figure legend. We focused on the sites that regulate the switch from translational repression (deadenylation) to activation. For CPEB1 a single phosphorylation site was characterized in the past (Mendez 2000 a, b). For CPEB4 multiple phosphorylation sites by Cdk1 and ERK1/2 were identified (Guillen-Boixet et al. 2016). For CPEB1 it is reported a bimodal regulation whereas for CPEB4 phosphorylation causes intermediate states as a factor of the number of phosphorylated residues. For CPEB4, the regulation was independent of the specific residue, but rather the result of the number of residues independently of the exact residues and directly related to the disruption of the aggregation by the negative charges. Now we identify that CPEB2 and 3 are phosphorylated by the same kinases at proline-directed sites (consequently with the specificity of the kinases). The distribution of the sites in the IDRs of CPEB2-3 is equivalent to that of CPEB4 as it is their effect on LLD assembly. We show that these mutations prevent in vivo and in vitro phosphorylation. It should be noted that the change of charge only takes place if the site is phosphorylated, otherwise the mutation would be neutral. Altogether we show now that the regulation of CPEB2,3 is identical to the described for CPEB4 (and therefore coordinated) while different from that of CPEB1.***

9. The analysis of the BioID and RIP-Seq datasets should be made publicly available.

***They are now public and can be found at https://github.com/BBIRBCF/CPEBs-RIPSeq-analysis and https://github.com/BBIRBCF/CPEBs-BioID-analysis.***

Minor
1. Their introduction should introduce Maskin, PARN, and GLD2 to the reader.
***We have included a brief description.***

2. Do the authors conclude that "ortholog-specific phenotypes when individually depleted" demonstrate CPEB family members have differential functions? Even the individual depletion of paralogs would not show this due to possible complications associated with redundancy.
***We apologize for the mistake, we meant “paralog-specific phenotypes”. As the reviewer mentions, the fact that individual paralog depletions display a phenotype, and specific for each paralog, indicates that they are not fully redundant.***

3. In light of Zaccara et al. 2020 Cell, do the authors conclude that the proximity of CPEB1 with YTHDF1 implicates CPEB1 in translational repression?
***The interplay between N6-methyladenosine (m6A), Cap-Ribose methylation and the poly(A) tail, as well as the possible contributions of CPEBs in coordinating these three events is currently under study in our laboratory. At this point we are not ready to propose a model. The link between CPEBs and deadenylation (through CCR4-NOT) is much stronger (and further supported by a companion submitted manuscript by Poetz et al.).***

4. The authors refer to HuR as HuR and ELAVL1 in their STRING analysis. This should be changed or explained to prevent confusion.
***We have unified the nomenclature used throughout the whole manuscript, in 4C ELAVL1 is now referred to as HuR.***

5. How do the authors reconcile their data on CPEB family members having proximity to distinct proteins (F4B) while present in the same complexes (F4D/SF5A)?
***As shown in the figure representing a possible CPEB-complex (see ‘Point by Point’ Figure 2), at an approximate scale, modeling the CCR4-NOT complex with the published positions of DDX6 and AGO (Ukleja et al. 2016) and a possible location of CPEB fused to BirA at the N- (red) or C- (orange) termini together with an “endogenous” CPEB (green). BirA biotinylates proteins in a radius of 10-15 nm (inner and outer dashed line circles in the figure), this would include only biotinylation of the closest complex components, whether or not they physically interact. It will not label other complexes independently binding the same mRNA. Weak or transient interactions are potentially detected by BioID. In contrast, co-IP would presumably immunoprecipitate the whole complex (which is 12-14 MDa; Figure 3A) including long-range components and intermediate proteins.***

6. What are the authors' interpretations of the results presented in SF6A and SF6B? Their conclusion should be explicit. For example, what were the observed differences?
***Superresolution/ultraexpansion microscopy showed intradroplet segregation between different CPEBs (Figure S6A – now S7A), while co-FRAP showed different exchange dynamics between the soluble and aggregated fractions (Figure S6B – now S7B). This would suggest that although individual CPEB-liquid-like condensates co-exist in the same droplets they maintain compartmentalized localization and properties.***

7. Authors must cite the work that shows progesterone promotes the CPEB1-switch from repression to activation.
***We have included the reference.***

8. Why are CPEB1, DDX6, and EIF4ENIF not abundant in lower fractions after size fractionation? Are these proteins only present in massive foci?
***The result would indicate that these proteins are not present in the cytoplasm as monomers/dimers but rather always part of large complexes (> 12-14 MDa).***

9. What proportion of CPEB is in liquid-like droplets? What is the concentration of CPEB in liquid-like droplets?
***The distribution of soluble vs LLD for each CPEB is in Figure 2C right panel. To the best of our knowledge, it is not technically possible to determine the concentration of CPEBs in the LLD in vivo.***

10. Do DDX6 and EIF4ENIF have roles in translational repression in CPEB activation complexes?
***From the literature and the results in this work, we would argue that these proteins are structural mRNP components, without specific functions in translational repression nor activation.***

11. The headings throughout the manuscript should state the conclusion of the following section. In addition, greater emphasis should be given to the narrative that CPEB family members form two distinct but overlapping subfamilies. Finally, the justification of each following technique should be made clear; together, these changes will reduce the misinterpretation that the authors have linked together disconnected datasets.
***We thank the reviewer for these suggestions. We have tried to improve the conceptual flow of the manuscript and we have therefore changed the headings throughout the manuscript.***

12. In the first section of results "Meiotic maturation as a model for coordinated expression and regulation of all four CPEBs", except for MS/MS, the authors showed the data only for CPEB2-3 and relied on the previously published data for CPEB1,4 to compare with. It would have been more convincing to see CPEB1 and CPEB4 in the same experiment, instead of referring to a 1994 paper to compare.
***We agree with the reviewer, but we were handicapped by the availability of antibodies. Our initial aim was to provide quantitative comparisons, but due to the limitations with antibodies and the fact that MS/MS is only partially quantitative, we have limited the analysis to bimodal (presence/absence) conclusions.***

13. The inputs in CPEB2 immunoprecipitation (Figure 1A and Figure S3-B) are not visible.
***Please, note that Figure 1A is not an immunoprecipitation but rather a b-isox chemical precipitation, commonly used to concentrate RNA-binding proteins that contain low-complexity and unstructured regions (Han et al., 2012; Kato et al., 2012). We used this approach to concentrate the low abundant endogenous CPEB2-3 to detect them by immunoblot. Due to their low abundance, and for the affinity of the available antibodies, endogenous CPEB2-3 can barely be detected in the non-concentrated samples. The amount equivalent to 50 oocytes was loaded in the b-isox precipitated lanes, compared to one oocyte in the input lanes.***

***In figure S3-B, CPEB2 overexpression levels were low, due to the intrinsic low stability of this protein. We have now replaced Figure S3-B with a replicate where CPEB2 can be clearly seen in the inputs.***

14. In Figure 5E, the authors are stating "We did not find any specific functional category for CPEB1, On the other hand, G1-S cell cycle control and TNF-alpha signaling were enriched among CPEB2-4 targets". The p-value for NOTCH1 signaling pathway enrichment is p<0.05 in CPEB1-targets and <0.01 in CPEB2-4 targets. Why there is a focus on G1-S and TNF-alpha pathways, even though they are less enriched (yet significant)?
***We apologize for not being clear. We wanted to emphasize the functional categories that were distinct for CPEB1 or CPEB2-4 targets. NOTCH is present in both groups and therefore we focused in the remaining significant categories.***

15. There are a couple of mislabeling in supplementary figures:
a. Figure S3-D IP-E figure: inversion of FLAG-Ubi with HA-GFP-CPEB2
***This was a mistake and has been corrected.***

b. Figure S6-A, if the FRAP is performed on the same confocal slides, then the labeling in S6-A panel 2 should be CPEB1-mCherry and CPEB2-GFP instead of CPEB2-mCherry and CPEB1-GFP, and in panel 3, should be CPEB3-GFP instead of CPEB2-GFP, otherwise, it doesn't make sense.
***Please, note that Figure S6A (now S7A) does not correspond to the FRAP but the UExM, whereby we looked at the co-localization of CPEB1 and CPEB2 in foci in an ultraexpansion/superresolution microscopy setting.***

**Second round of review**

**Reviewer 1**

The authors have addressed all my concerns.

**Reviewer 2**

I commend the authors for their efforts to address the critique. The manuscript is strengthened and I recommend publication.
